# Development and Psychometric Properties of the Multi-System Profile of Symptoms Scale in Patients with Rett Syndrome

**DOI:** 10.3390/jcm11175094

**Published:** 2022-08-30

**Authors:** Jatinder Singh, Federico Fiori, Mei Lin Law, Ruksana Ahmed, Shashidhar Ameenpur, Salah Basheer, Samiya Chishti, Rosie Lawrence, Mathilde Mastroianni, Abdolreza Mosaddegh, Paramala Santosh

**Affiliations:** 1 Department of Child and Adolescent Psychiatry, Institute of Psychiatry, Psychology and Neuroscience, King’s College London, London SE5 8AF, UK; 2 Centre for Interventional Paediatric Psychopharmacology and Rare Diseases (CIPPRD), South London and Maudsley NHS Foundation Trust, London SE5 8AZ, UK; 3 Centre for Interventional Paediatric Psychopharmacology (CIPP) Rett Centre, Institute of Psychiatry, Psychology and Neuroscience, King’s College London and South London and Maudsley NHS Foundation Trust, London SE5 8AZ, UK; 4 HealthTracker Limited, 76-78 High Street Medical Dental, High Street, Gillingham ME7 1AY, UK

**Keywords:** Rett Syndrome, Multi-System Profile of Symptoms Scale, PROM, eObsRO, item response theory, web-based, psychometric properties

## Abstract

*Background*: Rett Syndrome (RTT) is a rare, neurodevelopmental disorder characterised by a range of problematic symptoms. There is yet to be a robust instrument to adequately capture the range of disease severity across the lifespan. In this study, we aimed to develop and assess the validity of an RTT-specific electronic Observer Reported Outcome (eObsRO), the Multi-System Profile of Symptoms Scale (MPSS). *Methods*: The study was conducted in two phases. Phase 1 consisted of a systematic literature review, focus groups, expert feedback, and a pilot test of the new scale. Modifications were made based on preliminary analysis and feedback collected in the pilot phase. Phase 2 consisted of the validation of the questionnaire based on two samples (Sample 1, *n* = 18; Sample 2, *n* = 106). Participants were all parents or caregivers of individuals with RTT. *Results*: The MPSS consists of 12 validated sub-scales (mental health problems, autonomic problems, cardiac problems, communication problems, problems in social behaviour, problems in engagement, gastrointestinal problems, problems in motor skills, neurological problems, orofacial problems, respiratory problems, and sleep problems), which explore symptom frequency in the past month and a supplement to the scale consisting of five sub-scales (sensory problems, immune dysfunction and infection, endocrine problems, skeletal problems, and dermatological problems), which is designed to capture symptom changes over a longer time period. The frequency of symptoms was rated on a 10-point slider scale, which then was automatically transformed into a 0 to 5 Likert score. All 12 sub-scales showed strong internal consistency (α ≥ 0.700) and good stability, ranging from 0.707 to 0.913. Pearson’s correlation showed a statistically significant (r = 0.649) correlation between the MPSS and the Rett Syndrome Behaviour Questionnaire (RSBQ) total score and significant correlations between sub-scales with items that were presented in both the MPSS and RSBQ. *Conclusions*: The MPSS is a psychometrically validated eObsRO using the HealthTracker^TM^ platform and has the potential to be used in clinical trials.

## 1. Introduction

Rett Syndrome (RTT) is a pervasive neurodevelopmental disorder that predominantly affects females and is estimated to occur in approximately 1 in 10,000 live female births [1]. RTT was first described by Andreas Rett as a regressive disorder affecting very young girls who had early periods of typical development [2]. Prior to the discovery of potential biomarkers, the diagnostic criteria for RTT had been exclusive for females and mainly characterized by multiple developmental regressions, including rapid loss of behavioural, social, and psychomotor skills after a period of typical development in the first year of life [3]. Cases of RTT are attributed to mutations in the gene (*MECP2*) encoding X-linked methyl-CpG-binding protein 2 (*MECP2*) [4], which had since been identified in most cases of typical RTT [5,6]. While the location of the mutation on the X chromosome was formerly thought to be lethal in males, there had been increasing reports of males with RTT [7,8]. More than half of the male cases reported in a systematic review had *MECP2* gene mutations, although other genetic abnormalities were also present [8], making males more difficult to identify. Since the earliest descriptions of the clinical criteria in RTT [9,10], substantial progress has been made in the understanding of clinical manifestations of RTT variants [11]. Clinical criteria differ between the diagnosis of typical or atypical RTT [1].

The severity of RTT symptoms varies according to mutations within the *MECP2* gene, but severe functional impairments in motor and communication skills are usually present, requiring substantial care [12]. Moreover, RTT is commonly associated with debilitating comorbidities such as epilepsy, growth retardation, autonomic dysfunction, scoliosis, sleep disturbances, and adverse bone health, further exacerbating the challenges faced in the treatment and care of individuals with the disorder [12].

### Challenges for Clinical Trials

Considering the heterogeneity across individuals with RTT, effective treatment strategies are unlikely to be universally applicable. Still, the quality of life of patients can in-part be improved through the development of newer interventions [13] or potentially repurposed or exploratory agents in clinical trials. In disorders like RTT, clinical trials are faced with multiple challenges including variations across the mutational landscape and the developmental trajectory of the disorder. Although alternative clinical trial designs can be used to address heterogeneity in samples, some designs might be better than others to capture specific symptoms [14,15]. Studies on animal models have contributed substantially to the understanding of *MECP2* and potential therapeutic options, but similar results were not replicated in clinical trials [16,17,18]. Still, continued progress has been made since RTT was first discovered, with improved understanding of the disorder and strategies to accelerate clinical trials [19].

Research into RTT clinical trials has been hindered by the limitations in robust outcome measures [12,15,17,19]. Several pre-existing instruments used in RTT were available for multiple purposes, but no single instrument can adequately illustrate disease severity and heterogeneity across an individual’s lifespan [20]. Some instruments that have been used as outcome measures include the Quality-of-Life Inventory-Disability (QI-Disability) measure, which highlights important quality of life domains for children with intellectual disabilities across four diagnostic groups including RTT [21,22]. Similarly, several RTT-specific measures focused on a particular aspect of the heterogenous disease. The Motor Behavioural Assessment (MBA) originally consisted of 39 items to describe movement disturbances in RTT [23]. More recently, the number of items has been modified, and psychometric analysis suggests that the revised MBA would be useful when assessing clinical severity in RTT [24]. Other outcome measures such as the Rett Syndrome Motor Evaluation Scale [25] and the Rett Syndrome Gross Motor Scale [26] would also be helpful when assessing motor problems in Rett patients. The clinician-rated Clinical Global Impression (CGI) scales are used to rate patients’ global functioning before and after treatment in trials based solely on clinicians’ judgement and knowledge of a patient’s history [27]. Moreover, the RTT-specific version of the CGI offers the potential to capture changes to the seven core symptom domains in RTT [28]. The Rett Syndrome Behaviour Questionnaire (RSBQ) was developed to differentiate the disorder from other severe intellectual disabilities [29] and has been widely used as an outcome measure in clinical trials of patients with RTT [15]. However, recent publications suggest that the RSBQ may not adequately be able to capture clinically meaningful changes in RTT [30,31]. Electronic reported Clinical Outcome Assessments (eCOA) have gained momentum in recent years, and electronic Observer-Reported Outcomes (eObsRO) may offer another route to improve outcomes in clinical trials of rare diseases [32], especially in the paediatric population when used with other outcome measures [33].

Based on the complex profile of symptoms seen in RTT, comprehensive assessments need to be capable of capturing disease severity, changes in symptoms according to disease progression, and responses to treatments. Systematic approaches based on the assessment of emotional, behavioural, and autonomic dysregulation (EBAD) have been used to manage RTT patients in clinical settings [34,35,36]. These approaches provide clinicians and researchers with valuable information by merging the observed evaluation of emotional and behavioural dysregulation with objective measurements of autonomic functions. EBAD demonstrates high potential to provide tangible targets for treatment in clinical trials, especially in disorders with complex psychopathology such as RTT [15].

The Tailored Rett Intervention and Assessment Longitudinal (TRIAL) Database study [20] addresses the urgent need to develop a comprehensive, multisystem questionnaire, which can be linked to genetic information and physiological data. As part of the TRIAL Database study, a RTT-focused questionnaire was developed. This was initially called the Rett Evaluation of Symptoms and Treatments (REST) questionnaire to provide an adequate measure of disease severity across the lifespan (34). As it became clear that the instrument was a measure of multisystem profiling, which could be used across rare disorders, we described it as the Multi-System Profile of Symptoms Scale (MPSS). We present the validity and reliability of the MPSS in Rett syndrome and its correlation with the RSBQ.

## 2. Methods

**Figure 1:**
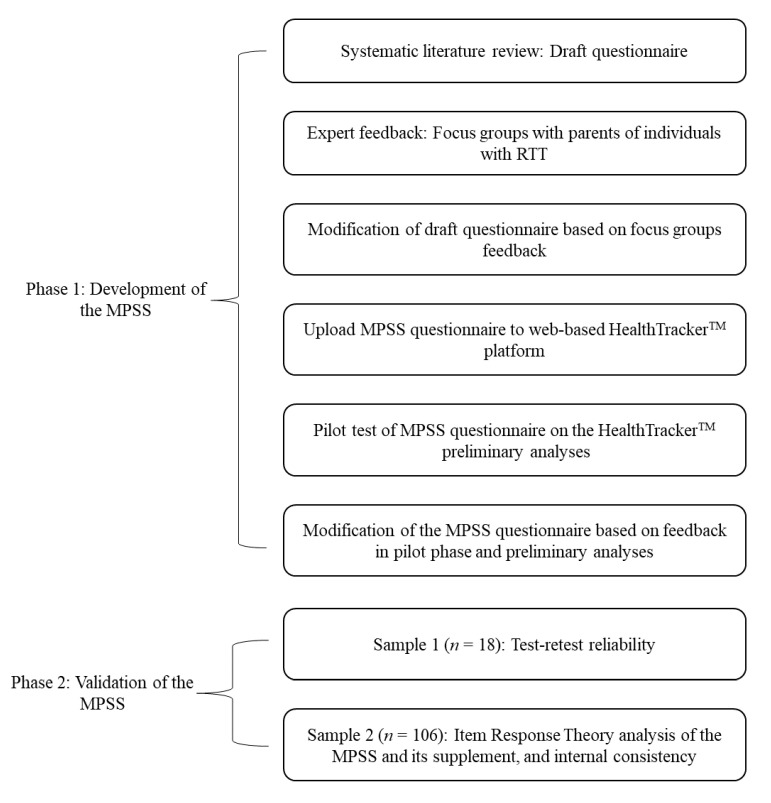
General overview of the development and validation of the Multi-System Profile of Symptoms Scale (MPSS) in patients with RTT.

Ethics approval was obtained from the NHS London-Bromley Research Ethics Committee (REC reference: 15/LO/1772) for the study. Figure 1 shows the general overview of the development and validation of the scale, which was conducted in two phases. Participants were recruited through parent-based charities such as Reverse Rett UK, the Centre for Interventional Paediatric Psychopharmacology (CIPP) Rett Centre, and clinicians who treat patients with RTT at the South London and Maudsley (SLaM) National Health Service (NHS) Foundation Trust in the U.K. Participants were parents or caregivers of individuals with a clinical diagnosis of RTT. Inclusion and exclusion criteria for each phase are described in the following sections. Informed consent was obtained from parents/caregivers at every phase of the study. As described in the protocol [20], the authors followed the guidance produced by the U.S. Food and Drug Administration (FDA) for the development of patient-reported outcome measures (PROM) [37] to develop the questionnaire. The COSMIN Study Design checklist was used to guide the design of the current study [38].

### 2.1. Phase 1: Development of the MPSS

#### 2.1.1. Concept Identification

In the preliminary stages, a systematic literature review was conducted to identify problematic symptoms in RTT [15]. The draft questionnaire incorporated important themes and elements that had been highlighted in previous studies [39,40]. Pre-existing questionnaires and diagnostic criteria [1] were also reviewed, including the RSBQ [29] and the modified version of the Rett Syndrome Severity Scale (RSSS) [41]. Expert clinicians with substantial experience in RTT were invited to provide feedback on the early drafts of the questionnaire. The draft version of the questionnaire was prepared based on the systematic review and expert feedback.

#### 2.1.2. Focus Groups

A consultant child and adolescent psychiatrist and two experienced researchers conducted focus groups as part of the qualitative development of the questionnaire in Phase 1. Parents/caregivers of individuals with a clinical diagnosis of RTT and clinicians who treat patients with RTT at SLaM were recruited. No age limit was imposed on individuals with RTT. Parents/caregivers who do not have a reasonable level of English were excluded as this was required to engage in the focus groups. Due to the nature of the focus groups, all participants were required to provide informed consent before participating. Two sessions of approximately 90 minutes duration each were conducted in English and were audio-recorded. The focus groups followed a semi-structured format, where some discussions were guided by paper copies of the draft version of the questionnaire and further discussions were led by open-ended questions to allow participants to share their feedback.

The first session focused on item generation. Participants were asked to review the draft version of the questionnaire and identify additional items that had not been mentioned in the focus groups. Based on the draft, participants were also asked to rate the importance of symptoms and key themes on a 10-point scale, where 0 = not important at all and 10 = very important. This rating was used to guide the order of symptoms in the questionnaire, where items deemed more important were placed first, followed by items of least importance. Feedback from this session was used to guide the amendments to the draft questionnaire. The second session focussed on discussions around the use of web-based questionnaires, where participants’ views and experiences were recorded. They were shown different examples of how the questionnaire can be presented on the HealthTracker^TM^ (described in the next section). Participants provided feedback on the appearance and functionality of each example and were asked to choose their preferred presentation. Participants’ preferences for the optimal web-based visualization of the questionnaire on the web-based HealthTracker^TM^ platform were recorded and their feedback incorporated into the tool review.

#### 2.1.3. Upload and Pilot Test of MPSS to HealthTracker^TM^

The HealthTracker^TM^ is an established web-based health monitoring platform, which had been used in multiple clinical and research settings. The HealthTracker^TM^ was used in the Suicidality: Treatment Occurring in Paediatrics (STOP) study [42,43], the Managing the Link and Strengthening Transition from Child to Adult Mental Health Care (MILESTONE) project [44], and the development and validation of a neuropsychiatric questionnaire [45]. Following the focus groups, a version of the MPSS was finalized and uploaded onto the HealthTracker^TM^ platform. Participants in the focus groups were asked to test this version of the questionnaire and provided feedback on user experience.

### 2.2. Scoring of the MPSS

The questions, response options, web-based presentation, and scoring were decided based on feedback from focus groups. Sub-scales in the questionnaire were categorized according to symptom domains. For example, the “autonomic problems” sub-scale would include all questions related to that symptom domain. All items in the MPSS asked about the frequency of a symptom. Based on focus group feedback, the responses to each question were provided using a 10-point slider scale for the ease of completion with answers ranging from 0 (almost never) to 10 (almost always). Subsequently, based on a discussion regarding psychometrics and the need for Likert-scale-based categories, especially when one is using the measure in clinical trials, the 0–10 raw scores were automatically transformed into 6 categories—0 being “not present”, 1–2 being “rarely”, 3–4 being “sometimes”, 5–6 being “quite often”, 7–8 being “very often”, and 9–10 being “all the time”, similar to other measures developed by the team [45]. The MPSS is composed of 12 sub-scales, and all questions had a recall period of one month. All sub-scales started with a screening item about the presence of any symptoms that were part of a specific symptom domain. If the answer to these screening items was negative, meaning that no symptoms were present, the entire sub-scale was skipped and the items that composed the sub-scale were automatically scored as not present.

#### Supplementary Section to the Scale

Certain symptoms of RTT, such as skeletal problems, change over a longer period. Based on clinical and parent feedback, these sub-scales needed a longer period of recall of six months. The Supplementary Section to the MPSS is valuable to capture other symptoms not captured in the main MPSS.

### 2.3. Phase 2: Analysis of the MPSS

#### 2.3.1. Recruitment and Eligibility Criteria

The MPSS was first administered to the initial recruitment of RTT participants on the HealthTracker^TM^. Parents/caregivers of individuals with a clinical diagnosis of RTT (of all ages) were recruited in Phase 2 of the study through clinicians or researchers within the SLaM NHS Foundation Trust. Parents/caregivers who did not have reasonable of English were excluded from the study as the questionnaire was only available in English at this stage. Similarly, parents/caregivers who were not able to complete the questionnaires were excluded from the study, but research assistants assisted parents/caregivers if required. Assistance included providing paper copies or help with completing questionnaires online if requested by parents/caregivers.

#### 2.3.2. Sample

Parents/caregivers were provided with information sheets and consent forms prior to recruitment. After returning signed informed consent forms, study participants were sent non-identifiable login credentials to access the questionnaires on the HealthTracker^TM^ platform. Two samples were derived from the participating parents/caregivers of individuals with RTT. Sample 1 (*n* = 18) consisted of parents/caregivers who completed the re-administration of the MPSS within four weeks after their baseline completion. Sample 2 (*n* = 106) consisted of all the parents/caregivers who completed baseline measurements of the MPSS. Parents/caregivers in this sample were also asked to complete the Rett Syndrome Behaviour Questionnaire (RSBQ).

#### 2.3.3. Analysis

Data from the focus groups in Phase 1 were organized using thematic and content analysis. Feedback from experts and parents was considered to develop the operating version of the MPSS, which was uploaded onto the HealthTracker^TM^ platform. To verify the feasibility of the questionnaire, user feedback was recorded during the pilot test in Phase 1 and during the data collection in Phase 2 to inform about future improvements to the scale.

Data collected in Phase 2 were analysed using SPSS version 28.0. As part of the ongoing verification of the scale on the HealthTracker^TM^, preliminary analyses were conducted during data collection to identify any inaccuracies or potential improvements to the scale.

#### 2.3.4. Item Response Theory

Item Response Theory (IRT) was used to validate the scale responses and estimate cut-offs for the items [46]. MPSS data were analysed using IRTPRO (version 6). A series of graded parameter response models was run. For each sub-scale, items were evaluated and subsequently removed if the items did not satisfy the model fitting criteria. The Akaike Information Criterion was used to judge the goodness of the model fit when an item was removed or a sub-scale was created. The final versions of the sub-scales were reviewed by experts in RTT and approved.

#### 2.3.5. Psychometric Analysis

Based on data collected in Phase 2, descriptive statistics were generated to characterize both samples. Sample 1 was used to assess the test–retest reliability of the sub-scales in the questionnaire through Intraclass Correlation Coefficients (ICCs), based on repeated completion of the scales within 4 weeks. Item Response Theory was performed on Sample 2. This sample was also used to assess the internal consistency of the measures, which are reported using Cronbach’s alpha.

Pearson’s correlation was performed between sub-scales from the MPSS with sub-scales from the RSBQ. The RSBQ was used because it has been validated and used in clinical trials in patients with RTT [15]. The analysis focussed on the relevant sub-scales and their comparison between the MPSS and RSBQ. This analysis assessed the ability of parts of the MPSS to capture symptoms that are also measured by the RSBQ.

## 3. Results

### 3.1. Phase 1: Qualitative Development of the Multi-System Profile of Symptoms Scale 

Clinicians, parents, and caregivers in Phase 1 of the study agreed that the presentation of symptoms according to key themes or symptom domains needed to be inclusive, such that the questionnaire remained relevant to all individuals with RTT across their lifespans. Parents and caregivers also specifically requested clear descriptions of symptoms being assessed as part of the questions to reduce ambiguity. They were supportive of the approaches to reduce the time taken to complete the questionnaires. Firstly, each sub-scale was presented per web page, with broad descriptions of symptoms relevant to each symptom domain. Using a branching methodology, only the relevant sub-scale items were presented. The web-based presentation of the MPSS on the HealthTracker^TM^ was positively received by parents and caregivers in the focus groups.

### 3.2. Preliminary Validation in Pilot Phase

#### Item Response Theory Analyses

**Figure 2:**
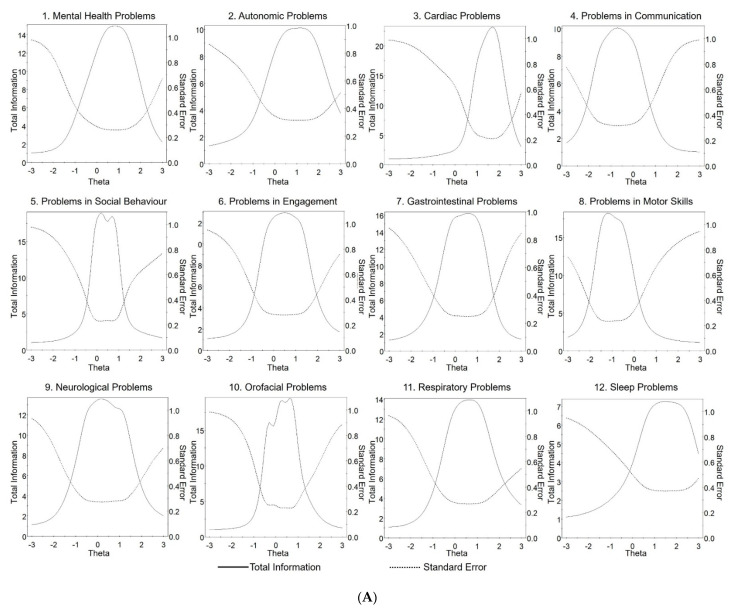

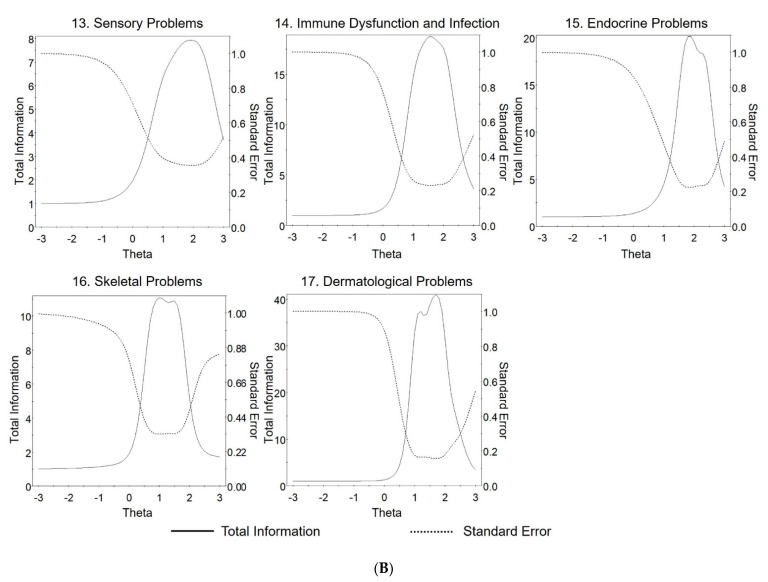
(**A**) Item characteristic curves for the sub-scale scores in the Multi-System Profile of Symptoms Scale (MPSS). (**B**) Item characteristic curves for the sub-scale scores in the Multi-System Profile of Symptoms Scale (MPSS) Supplement.

The slope and location parameters denoted by “a” and “b”, respectively, for the MPSS and its Supplement are presented in Table 1 and Table 2. The slope parameter reflects item discrimination and describes how well the items on the MPSS identified patients at different levels of the latent trait (represented by theta) being examined. Some of the items in the MPSS (Table 1) and its Supplement (Table 2) had higher slope values than others, indicating better discrimination. The location parameter reflects the difficulty of answering the items in the MPSS correctly. The Chi-squared and theta values for each item in the MPSS and its Supplement are shown in Supplementary Tables S1 and S2 (A and B). Figure 2A,B show the characteristic curves for the MPSS sub-scale total scores and describe the relationship between the responses and the sub-scales pictorially. Item curve characteristics for the MPSS are presented in Supplementary Figure S1 (A and B). After the IRT was performed, five items (excessive salivation, bruxism, lack of appetite, pain, and fever) did not reach the validation criterion. Despite this, these items were deemed to be clinically relevant for patients with RTT, and it was therefore decided to retain these as an additional sub-scale labelled “other problems”, recognising that this sub-scale has not been validated.

### 3.3. Phase 2: Validation of the MPSS 

Sample 1 consisted of 18 parents/caregivers of individuals with RTT (17 females, 1 male, mean age = 17.67 years, SD = 8.04, age range = 2.12–34.27 years). Participants were classified as Caucasian (94.4%) and of Other Mixed background (5.6%). This sample comprised participants who completed a re-administration of the MPSS within 4 weeks after their baseline completion. Data from this sample were used to test the time stability, or test–retest reliability, of the MPSS.

Sample 2 consisted of 106 parents/caregivers of individuals with RTT (105 females, 1 male, *M*_age_ = 16.71 years, SD = 11.20, age range = 2.11–51.96 years). Forty-one (41) parents of this sample also completed the baseline measurements of the MPSS and the RSBQ. Data were used to illustrate the profile of common symptoms in RTT and assess the internal consistency of the sub-scales and correlation with the RSBQ.

#### 3.3.1. Profile of Common Problems in RTT

**Figure 3:**
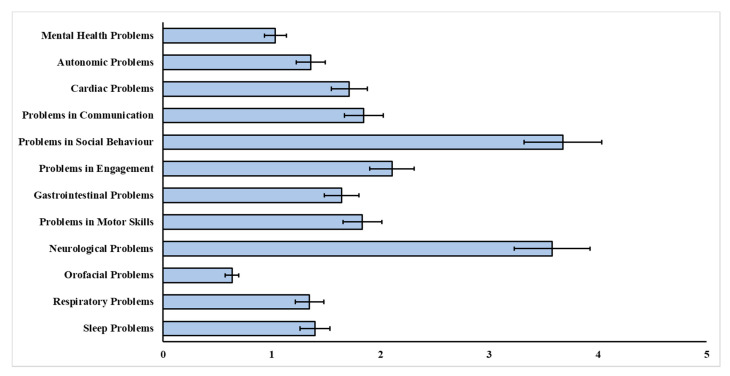
Mean sub-scale scores of the MPSS in Rett syndrome (*n* = 106). Notes: error bars are presented as the standard error.

To identify common symptoms in RTT, the number of problem domains rated as “present” in the MPSS are shown in Table 3. Figure 3 illustrates the mean scores of the MPSS sub-scales in the RTT sample (Sample 2, *n* = 106). These findings further supported the items identified by parents/caregivers and clinicians in the focus groups.

#### 3.3.2. Internal Consistency

Cronbach’s alpha measures were calculated for each sub-scale in the MPSS and its Supplement (Table 4). The results showed evidence of internal consistency across all sub-scales. All 12 sub-scales of the MPSS had alpha coefficients ≥0.700, indicating strong internal reliability, but not for a couple of the Supplementary sub-scales (skeletal problems (α = 0.385); sensory problems (α = 0.516)), which only showed modest internal consistency, indicating low to moderate internal reliability.

#### 3.3.3. Intraclass Correlation Coefficient

Test–retest reliability was assessed using intraclass correlation coefficients between the sub-scales’ total scores at the first and second administration (*M*_days_ = 15.21, *SD* = 8.09, range 7–30 days). The results showed good temporal stability for all 12 sub-scales of the MPSS (Table 4).

#### 3.3.4. Pearson’s Correlation

In this analysis, we used the proposed cut-off value of *r* > 0.2 to assess the strength of correlation [47]. The result showed a statistically significant (*r* = 0.649) correlation between the MPSS and RSBQ total score. We measured correlations between sub-scales that measured similar constructs in the MPSS and RSBQ. The “mental health problems” MPSS sub-scale was significantly correlated with the RSBQ “general mood” (*r* = 0.671) sub-scale. Similarly, there were significant correlations between “autonomic problems” (MPSS) and “fear anxiety” (RSBQ) (*r* = 0.425), “respiratory problems” (MPSS) and “breathing problems” (RSBQ) (*r* = 0.897), and “problems in communication” (MPSS) and “repetitive face movements” (RSBQ) (*r* = 0.323). 

“Problems in social behaviour” (*r* = 0.309) and “problems in engagement” (*r* = 0.367) MPSS sub-scale scores also showed significant correlations with the RSBQ “body rocking expressionless face” sub-scale. Finally, the RSBQ “hand behaviour” demonstrated significant correlations with the “problems in motor skills” (*r* = 0.328) and “neurological problems” (*r* = 0.346) MPSS sub-scales.

## 4. Discussion

The MPSS was developed as a comprehensive eObsRO, which is based on parent- or caregiver-rated symptoms of rare diseases. In this paper, we reported the results of the development and validation of the MPSS and presented its validation in RTT. The results showed that the MPSS is a reliable and valid instrument, which can be used longitudinally to illustrate disease progression in RTT. Supported by the functionalities of the HealthTracker^TM^ platform, the MPSS is now validated for web-based completion, which extends its use to any individuals with Internet-connected devices. Further, the questionnaire can also be administered by clinicians or researchers who can access it on mobile devices to allow the real-time capture and storage of data.

Improvements to the MPSS were implemented throughout this study by the ongoing collection of user feedback. These approaches made it possible to develop a validated questionnaire, which remains comprehensive, clinically relevant, and meaningful to families. All items ask about symptom frequency on a six-category Likert scoring, which is an eObsRO that was easy to complete by parents and caregivers. This validated RTT-specific eObsRO has the potential to be used in clinical trials. The overall conclusion we reached about the MPSS based on the IRT analysis is that the scale is a psychometrically valid measure of symptoms in patients with RTT. Both the model and item fit indexes were acceptable. Each item had a substantive link to the latent trait. Items had slope parameters indicating they were able to differentiate respondents with different levels of symptoms with some items being able to discriminate better than others. This finding is important because it suggests that individual sub-scales and their items can themselves be used if a change in a specific set of symptoms was being examined. In summary, 13 symptom domains were identified in the MPSS, of which 12 were psychometrically validated. The supplement to the scale identified an additional five symptom domains that can capture symptom changes over a longer duration. When viewed collectively, the scale was able to collect data in RTT in the current sample across different ages to profile both short-term and long-term symptom changes.

In comparison with existing outcome measures used in RTT, the MPSS was able to capture multisystem symptomatology in a single instrument. During the qualitative development phase of the MPSS, parents and clinicians highlighted several important symptoms that are not typically assessed. The Clinical Global Impression (CGI) scale is a summary of individuals’ global functioning based on clinicians’ knowledge of patients’ disease progression, while the MBA is concerned with specific motor functions and behaviour. The RSBQ is one of the commonly used RTT scales available but does not cover all the domains that the MPSS captures. We acknowledge the discussion about the psychometric properties of the RSBQ [30,31,48] and are cognisant that, given the complex organic features of RTT, it is unlikely that a single outcome measure will be suited, and its development will need to adopt a holistic approach involving multiple stakeholders [19]. Nevertheless, the MPSS captures the major problems in RTT and can potentially be used in clinical trials and in measuring symptoms longitudinally, in particular if a broad profile of symptoms needs to be evaluated. Future work on the MPSS will help to assess its utility in clinical trials and symptom changes across the disorder trajectory.

The present study reports the development, and demonstration of acceptable psychometric properties of the RTT-specific eObsRO, the MPSS, and that it is acceptable to parents, caregivers and other informants, and its items are feasible for use in RTT. The ongoing data collection will generate a larger sample for future studies, which will permit the stratification of data by age and clinical stages of the disease. The use of the MPSS sub-scales that can capture symptom changes quickly will ideally encourage its use in clinical trials. Given the complexity of the disorder and the lack of appropriate instruments validated for use in RTT, the MPSS offers a useful outcome measure with broad digital applications, which makes it practical from the perspective of both clinical and research settings.

## Figures and Tables

**Table 1:** Multi-System Profile of Symptoms Scale—Item Response Theory analysis.

**Mental Health Problems**	***a***	***s.e.***	***b*_1_**	***s.e.***	***b*_2_**	***s.e.***	***b*_3_**	*****s.e.*****	***b*_4_**	***s.e.***	***b*_5_**	***s.e.***	***X* ^2^**	***d.f.***	**Probability**
Aggression	2.69	0.56	0.43	0.14	0.73	0.15	0.95	0.17	1.12	0.19	1.62	0.25	26.4	17	0.0674
Self-Injury	2.8	0.64	0.51	0.14	0.76	0.16	1.02	0.18	1.42	0.23			16.24	17	0.5081
Screaming	2.5	0.5	−0.04	0.14	0.31	0.14	0.49	0.15	1.07	0.19	1.48	0.24	25.54	25	0.4341
Fears	2.41	0.5	0.3	0.14	0.76	0.16	0.91	0.18	1.55	0.25	1.74	0.28	33.87	19	0.019
Agitation	3.14	0.64	−0.67	0.16	−0.38	0.14	0.05	0.13	0.49	0.14	1.2	0.19	41.83	27	0.0341
Panic Attacks	2.15	0.41	−0.08	0.15	0.34	0.15	0.61	0.16	1.19	0.21	1.74	0.28	39.84	26	0.0403
Low Mood	1.82	0.37	−0.17	0.16	0.38	0.16	0.83	0.19	1.34	0.25	1.74	0.31	43.26	32	0.0881
**Autonomic Problems**	***a***	***s.e.***	***b*_1_**	***s.e.***	***b*_2_**	***s.e.***	***b*_3_**	***s.e.***	***b*_4_**	***s.e.***	***b*_5_**	***s.e.***	***X* ^2^**	***d.f.***	**Probability**
Temperature Changes	2.86	0.6	−0.21	0.14	0.38	0.14	0.77	0.15	1.3	0.19	1.73	0.25	46.24	29	0.0221
Sweating	2.54	0.57	0.4	0.14	1.02	0.17	1.31	0.2	1.57	0.24	2.22	0.37	32.3	20	0.0401
Pupillary Changes	2.68	0.56	0.21	0.14	0.68	0.15	0.83	0.16	1.37	0.2	1.74	0.25	50.64	25	0.0018
Urination	1.7	0.4	0.54	0.17	1.49	0.28	1.67	0.31	2.16	0.41	2.55	0.51	28.02	22	0.1744
Cold Limbs	1.26	0.28	−1.71	0.37	−1.32	0.31	−1.11	0.28	−0.3	0.2	0.56	0.21	44.72	33	0.0835
Breath-Holding During Sleep	1.2	0.32	0.61	0.22	1.26	0.31	1.9	0.43	3.15	0.77	3.78	0.99	36.36	22	0.0277
Shallow Breathing	1.05	0.26	0.02	0.22	0.65	0.24	1.05	0.29	1.71	0.41	2.4	0.56	26.18	32	0.7562
Diarrhoea	0.8	0.25	0.21	0.27	1.84	0.54	2.25	0.65	3.63	1.07	4.36	1.34	34.56	26	0.1212
**Cardiac Problems**	***a***	***s.e.***	***b*_1_**	***s.e.***	***b*_2_**	***s.e.***	***b*_3_**	***s.e.***	***b*_4_**	***s.e.***	***b*_5_**	***s.e.***	***X* ^2^**	***d.f.***	**Probability**
Irregular Heartbeat	4.76	1.78	1.03	0.16	1.32	0.18	1.55	0.21	1.75	0.24	1.9	0.27	20.74	7	0.0042
Fainting	4.06	1.57	1.67	0.23	1.89	0.27	2.12	0.34	2.33	0.42					
Sudden Changes in Heart Rate	4.72	1.51	0.97	0.15	1.21	0.17	1.36	0.19	1.58	0.22	1.89	0.27	19.93	8	0.0106
Postural Change	2.67	0.87	1.33	0.22	1.5	0.25	1.78	0.3	2.04	0.37			8.65	8	0.3747
Rapid Heartbeat	1.79	0.45	−0.18	0.17	0.34	0.17	0.66	0.19	1.24	0.25	2.05	0.4	14.34	11	0.214
**Problems in Communication**	***a***	***s.e.***	***b*_1_**	***s.e.***	***b*_2_**	***s.e.***	***b*_3_**	***s.e.***	***b*_4_**	***s.e.***	***b*_5_**	***s.e.***	***X* ^2^**	***d.f.***	**Probability**
Following Commands	3.69	0.93	−1.52	0.23	−0.94	0.17	−0.67	0.15	−0.37	0.14	0.03	0.13	21.15	21	0.4512
Understanding Words	2.89	0.65	−1.18	0.2	−0.75	0.16	−0.39	0.14	0.23	0.14	0.49	0.15	14.25	25	0.9574
Non-Verbal Communication	2.01	0.43	−1.47	0.26	−0.95	0.2	−0.67	0.18	−0.25	0.16	0.01	0.16	35.53	23	0.0459
Vocalisation	1.69	0.43	−2.03	0.37	−1.92	0.35	−1.73	0.32	−1.29	0.25	−0.82	0.19	21.67	17	0.1974
**Problems in Social Behaviour**	***a***	***s.e.***	***b*_1_**	***s.e.***	***b*_2_**	***s.e.***	***b*_3_**	***s.e.***	***b*_4_**	***s.e.***	***b*_5_**	***s.e.***	***X* ^2^**	***d.f.***	**Probability**
Being Touched or Held	1.59	0.39	0.54	0.17	1.15	0.24	1.56	0.31	1.89	0.38	2.4	0.5	34.32	23	0.0605
Repetitive Behaviour	2.61	0.54	−0.21	0.14	0.09	0.13	0.15	0.13	0.4	0.13	0.77	0.15	30.12	20	0.0678
Routines	6.77	3.24	−0.07	0.12	0.18	0.12	0.3	0.11	0.64	0.12	0.86	0.13	41.96	20	0.0028
Eye Contact	1.67	0.35	−0.28	0.19	−0.07	0.18	0.14	0.18	0.74	0.2	1.25	0.25	34.27	23	0.0612
**Problems in Engagement**	***a***	***s.e.***	***b*_1_**	***s.e.***	***b*_2_**	***s.e.***	***b*_3_**	***s.e.***	***b*_4_**	***s.e.***	***b*_5_**	***s.e.***	***X* ^2^**	***d.f.***	**Probability**
Disengagement	3.06	0.63	−0.17	0.16	0.33	0.14	0.65	0.14	1	0.16	1.48	0.2	43.86	26	0.0156
Lethargy	4.29	1.09	−0.35	0.16	0.05	0.14	0.41	0.13	0.77	0.14	1.24	0.17	43.75	25	0.0115
Alertness	1.89	0.37	−0.19	0.18	0.13	0.17	0.55	0.17	1	0.2	1.44	0.25	30.64	31	0.4856
Energy Level	1.93	0.44	−0.01	0.17	0.34	0.16	0.55	0.16	0.81	0.17	1.28	0.22	47.95	30	0.02
Drowsiness	1.51	0.37	−0.29	0.21	0.01	0.19	0.33	0.18	0.55	0.18	0.86	0.21	51.91	30	0.0078
Dry Mouth	0.9	0.26	0.25	0.25	1.36	0.39	2.19	0.58	3.06	0.81	3.69	1.01	34.89	30	0.246
**Gastrointestinal Problems**	***a***	***s.e.***	***b*_1_**	***s.e.***	***b*_2_**	***s.e.***	***b*_3_**	***s.e.***	***b*_4_**	***s.e.***	***b*_5_**	***s.e.***	***X* ^2^**	***d.f.***	**Probability**
Abdominal Pain	4.2	0.91	−0.4	0.16	−0.06	0.16	0.42	0.19	0.78	0.21	1.12	0.24	37.76	26	0.0636
Pain after Meal	4.04	1.11	−0.06	0.16	0.36	0.2	0.73	0.24	1	0.27	1.35	0.31	34.13	24	0.0822
Abdominal Bloating	2.53	0.59	−0.66	0.16	−0.01	0.17	0.3	0.2	0.68	0.24	1.01	0.28	33.26	35	0.5535
Constipation	2.26	0.43	−0.86	0.21	−0.27	0.18	−0.06	0.18	0.36	0.18	0.74	0.2	55.18	32	0.0066
Acid Reflux	1.48	0.36	−0.27	0.19	0.36	0.22	0.68	0.26	1.24	0.35	1.66	0.43	54.16	35	0.0203
Toileting	1.2	0.29	−1.55	0.35	−0.79	0.24	−0.36	0.21	0.21	0.23	0.48	0.27	48.58	36	0.0784
**Problems in Motor Skills**	***a***	***s.e.***	***b*_1_**	***s.e.***	***b*_2_**	***s.e.***	***b*_3_**	***s.e.***	***b*_4_**	***s.e.***	***b*_5_**	***s.e.***	***X* ^2^**	***d.f.***	**Probability**
Gross Motor Skills	4.38	1.13	−1.45	0.2	−1.18	0.17	−1.05	0.16	−0.54	0.13	−0.18	0.12	28.64	21	0.1227
Fine Motor Skills	4.62	1.26	−1.52	0.21	−1.4	0.19	−1.24	0.18	−0.94	0.15	−0.57	0.13	27.65	15	0.0238
Clumsiness	2.72	0.61	−1.63	0.25	−1.16	0.19	−1.12	0.19	−0.87	0.16	−0.48	0.14	16.52	17	0.4885
Gait and Balance	2.14	0.44	−1.5	0.26	−1.02	0.2	−0.78	0.18	−0.34	0.15	0.05	0.15	39.9	25	0.0298
Stereotypic Hand Movements	1.09	0.3	−3.42	0.87	−2.42	0.59	−1.81	0.45	−1.3	0.34	−0.64	0.24	32.43	25	0.1456
Writhing Limb Movements	1.21	0.29	−0.55	0.22	−0.02	0.2	0.13	0.2	0.37	0.21	0.76	0.25	27.88	26	0.3661
**Neurological Problems**	***a***	***s.e.***	***b*_1_**	***s.e.***	***b*_2_**	***s.e.***	***b*_3_**	***s.e.***	***b*_4_**	***s.e.***	***b*_5_**	***s.e.***	***X* ^2^**	***d.f.***	**Probability**
Muscle Spasms	4.11	0.9	−0.46	0.16	−0.01	0.14	0.18	0.14	0.55	0.14	1.14	0.17	40.18	26	0.0373
Muscle Stiffness	2.77	0.51	−0.96	0.2	−0.29	0.16	−0.01	0.15	0.37	0.15	1.11	0.18	39.16	33	0.2122
Abnormal Muscle Movements	2.64	0.51	−0.57	0.19	−0.09	0.16	0.22	0.15	0.7	0.16	1.4	0.21	50.96	30	0.0098
Tremors	2.32	0.44	−0.45	0.16	0.36	0.17	0.61	0.18	0.9	0.2	1.43	0.26	34.07	30	0.2772
Fasciculations	1.34	0.3	−0.05	0.2	0.7	0.21	1.04	0.24	1.98	0.38	2.57	0.5	42.03	34	0.1616
Seizures	1.16	0.27	−0.41	0.22	0.42	0.23	1.07	0.31	1.44	0.36	2.41	0.56	41.26	34	0.1826
**Orofacial Problems**	***a***	***s.e.***	***b*_1_**	***s.e.***	***b*_2_**	***s.e.***	***b*_3_**	***s.e.***	***b*_4_**	***s.e.***	***b*_5_**	***s.e.***	***X* ^2^**	***d.f.***	**Probability**
Chewing	6.46	3.42	−0.35	0.13	0.16	0.12	0.27	0.12	0.61	0.12	0.77	0.13	23.12	19	0.2314
Swallowing	2.89	0.55	−0.23	0.15	0.41	0.14	0.63	0.15	0.78	0.15	1.04	0.18	32.88	24	0.1064
Tongue Mobility	2.83	0.61	0.09	0.14	0.49	0.14	0.6	0.14	0.95	0.16	1.31	0.2	33.98	25	0.1079
Mouth Closure	1.46	0.32	0	0.18	0.5	0.19	0.8	0.21	1.1	0.25	1.4	0.29	49.39	29	0.0105
**Respiratory Problems**	***a***	***s.e.***	***b*_1_**	***s.e.***	***b*_2_**	***s.e.***	***b*_3_**	***s.e.***	***b*_4_**	***s.e.***	***b*_5_**	***s.e.***	***X* ^2^**	***d.f.***	**Probability**
Over-breathing	3.44	0.72	−0.09	0.15	0.25	0.14	0.46	0.14	0.75	0.15	1.06	0.17	30.51	25	0.2054
Air Swallowing	3.1	0.63	−0.15	0.15	0.21	0.15	0.47	0.15	0.99	0.17	1.36	0.2	32.86	27	0.2012
Breath-Holding When Awake	2.54	0.48	−0.46	0.17	−0.11	0.16	0.29	0.15	0.67	0.16	1.12	0.2	32.85	30	0.3281
Hyperventilation	2.03	0.38	−0.41	0.18	0.05	0.16	0.46	0.17	1.02	0.2	1.59	0.26	59.41	34	0.0045
Air-Puffing	1.89	0.38	0.16	0.17	0.54	0.17	0.89	0.19	1.38	0.24	2.11	0.36	29.99	28	0.3652
Gasping	1.51	0.33	0.14	0.18	0.74	0.2	1.04	0.23	1.49	0.29	2.07	0.4	37.76	31	0.1871
Apnoea	1.48	0.45	1.01	0.22	2.07	0.44	2.4	0.54	3.23	0.83			19.89	15	0.1759
Cyanosis	1.23	0.4	1.18	0.28	1.82	0.44	2.59	0.67	3.1	0.84	3.71	1.09	20.72	15	0.1455
**Sleep Problems**	***a***	***s.e.***	***b*_1_**	***s.e.***	***b*_2_**	***s.e.***	***b*_3_**	***s.e.***	***b*_4_**	***s.e.***	***b*_5_**	***s.e.***	***X* ^2^**	***d.f.***	**Probability**
Nightmares and Night Terrors	2.73	0.57	0.77	0.16	1.29	0.21	1.7	0.27	2.11	0.35	2.51	0.46	16.7	13	0.213
Night-Sweats	2.64	0.75	0.76	0.16	1.26	0.21	1.56	0.26	2.1	0.36	2.46	0.46	17.32	15	0.2993
Morning Wakefulness	1.41	0.38	0.21	0.18	1.21	0.26	1.47	0.31	2.02	0.42	2.55	0.55	25.2	20	0.1933
Clamminess	1.32	0.34	−0.25	0.2	0.67	0.21	1	0.25	1.49	0.33	2.3	0.49	42.25	27	0.031
Insomnia	1.23	0.34	−0.15	0.21	0.46	0.2	0.58	0.21	1.55	0.36	2.15	0.5	23.81	26	0.5881

Abbreviations: *d.f.* (degrees of freedom); *s.e.* (standard error); *X*^2^ (Chi-squared). Notes: A Graded Parameter Response Model was run. Columns *a* and *s.e.* describe the slope estimate. Columns *b*_1_ to *b*_5_ *s.e.* correspond to the single-item thresholds (location parameters). The Chi-squared (*X*^2^), *d.f.*, and probability are reported in the last three columns. Empty squares reflect those items that did not have anyone scoring at this level.

**Table 2:** Multi-System Profile of Symptoms Scale Supplement—Item Response Theory analysis.

**Sensory Problems**	***a***	***s.e.***	***b*_1_**	***s.e.***	***b*_2_**	***s.e.***	***b*_3_**	***s.e.***	***b*_4_**	***s.e.***	***b*_5_**	***s.e.***	***X* ^2^**	***d.f.***	**Probability**
Olfactory Function	2.66	1.1	1.56	0.29	2.25	0.48	2.42	0.55					14.96	3	0.0018
Auditory Function	3.38	2.51	1	0.18	1.59	0.29	1.65	0.31	2	0.43	2.28	0.56	14.39	7	0.0445
Visual Function	1.96	0.64	0.89	0.2	1.68	0.33	1.87	0.37	2.52	0.56			20.86	9	0.0133
**Immune Dysfunction and Infection**	***a***	***s.e.***	***b*_1_**	***s.e.***	***b*_2_**	***s.e.***	***b*_3_**	***s.e.***	***b*_4_**	***s.e.***	***b*_5_**	***s.e.***	***X* ^2^**	***d.f.***	**Probability**
Infections	5.07	2.22	0.98	0.15	1.13	0.15	1.51	0.2	1.62	0.21	2.03	0.31	24.8	6	0.0004
Respiratory Infections	4.18	1.38	1.31	0.18	1.62	0.23	1.68	0.24	2.13	0.35			7.18	3	0.0663
Urinary Tract Infections	2.68	0.84	1.35	0.22	2.01	0.36	2.66	0.59					9.75	4	0.0448
Food Intolerance	2.73	0.85	1.19	0.2	1.53	0.25	1.8	0.3	2.09	0.38			24.57	7	0.0009
**Endocrine Problems**	***a***	***s.e.***	***b*_1_**	***s.e.***	***b*_2_**	***s.e.***	***b*_3_**	***s.e.***	***b*_4_**	***s.e.***	***b*_5_**	***s.e.***	***X* ^2^**	***d.f.***	**Probability**
Puberty	3.29	1.6	1.48	0.34	1.83	0.43	2.02	0.49	2.35	0.61			8.31	2	0.0157
Menstruation	1.78	0.81	1.39	0.39	1.69	0.47	1.91	0.54	2.21	0.65			11.83	5	0.0372
Growth	6.35	1.85	1.67	0.34	1.76	0.36	1.96	0.41	2.37	0.53					
Hormonal Problems	2.84	1.11	1.85	0.45	2.08	0.53	2.34	0.64					5.5	2	0.0637
**Skeletal Problems**	***a***	***s.e.***	***b*_1_**	***s.e.***	***b*_2_**	***s.e.***	***b*_3_**	***s.e.***	***b*_4_**	***s.e.***	***b*_5_**	***s.e.***	***X* ^2^**	***d.f.***	**Probability**
Scoliosis	1.33	0.52	0.91	0.33	1.25	0.39	1.88	0.55	2.28	0.68	2.62	0.8	22.47	12	0.0325
Fractures and Osteopenia	0.98	0.75	3.1	1.93	5.2	3.6							3.29	2	0.1937
Joint Problems	5.48	0.54	0.73	0.11	0.98	3.45	1.14	5.07	1.47	2.69	1.66	0.8	23.99	10	0.0076
**Dermatological Problems**	***a***	***s.e.***	***b*_1_**	***s.e.***	***b*_2_**	***s.e.***	***b*_3_**	***s.e.***	***b*_4_**	***s.e.***	***b*_5_**	***s.e.***	***X* ^2^**	***d.f.***	**Probability**
Skin Rashes	5.63	2.12	1.11	0.19	1.56	0.25	1.69	0.28	1.91	0.32	2.33	0.43	9.56	4	0.0484
Skin Texture	8.19	1.48	1.02	0.18	1.2	0.21	1.51	0.25	1.7	0.28	1.88	0.3	10.79	4	0.029
Skin Discoloration	4.72	0.7	1.08	0.18	1.25	0.19	1.34	0.26	1.66	0.27	1.83	0.29	5.95	6	0.43
Other Skin Problems	3.01	1.04	1.78	0.31	2.06	0.43	2.58	0.67					3.49	2	0.1762

Abbreviations: *d.f.* (degrees of freedom); *s.e.* (standard error); *X*^2^ (Chi-squared). Notes: A Graded Parameter Response Model was run. Columns *a* and *s.e.* describe the slope estimate. Columns *b*_1_ to *b*_5_ *s.e.* correspond to the single-item thresholds (location parameters). The Chi-squared (*X*^2^), *d.f.*, and probability are reported in the last three columns. Empty squares reflect those items that did not have anyone scoring at this level.

**Table 3:** Frequency and relative percentages of symptoms collected in Phase 2.

Sub-Scales	Frequency (*n* = 106)	Percentage
Mental Health Problems	89	84.0
Autonomic Problems	97	91.5
Cardiac Problems	61	57.5
Problems in Communication	101	95.3
Problems in Social Behaviour	73	68.9
Problems in Engagement	92	86.8
Gastrointestinal Problems	97	91.5
Problems in Motor Skills	104	98.1
Neurological Problems	95	89.6
Orofacial Problems	77	72.6
Respiratory Problems	87	82.1
Sleep Problems	87	82.1
Sensory Problems	34	32.1
Immune Dysfunction and Infection	27	25.5
Endocrine Problems	23	21.7
Skeletal Problems	41	38.7
Dermatological Problems	23	21.7

**Table 4:** Cronbach’s alpha and intraclass correlation coefficient.

Sub-Scales	Intraclass Correlation Coefficient (Sample 1)	Cronbach’s Alpha (Sample 2)	No. of Items
**MPSS Domains**			
Mental Health Problems	0.881	0.881	7
Autonomic Problems	0.707	0.792	8
Cardiac Problems	0.913	0.802	5
Problems in Communication	0.734	0.808	4
Problems in Social Behaviour	0.471	0.773	4
Problems in Engagement	0.709	0.839	6
Gastrointestinal Problems	0.861	0.854	6
Problems in Motor Skills	0.769	0.820	6
Neurological Problems	0.871	0.851	6
Orofacial Problems	0.743	0.808	4
Respiratory Problems	0.844	0.846	8
Sleep Problems	0.745	0.697	5
**Supplementary Domains**			
Sensory Problems		0.516	3
Immune Dysfunction and Infection		0.716	4
Endocrine Problems		0.664	4
Skeletal Problems		0.385	3
Dermatological Problems		0.845	4

## Data Availability

Reasonable requests for data for this study can be made by contacting the corresponding author.

## Supplementary Materials

The following Supporting Information can be downloaded at: https://www.mdpi.com/article/10.3390/jcm11175094/s1, Table S1 (A and B): Multi-System Profile of Symptoms Scale—Marginal fits (X^2^) and Standardized Local Dependence X^2^ Statistics. Table S2 (A and B): Multi-System Profile of Symptoms Scale—Theta Scores. Figure S1 (A and B): Multi-System Profile of Symptoms Scale—Item Curve Characteristics.

## References

[1] Neul J.L., Kaufmann W.E., Glaze D.G., Christodoulou J., Clarke A.J., Bahi-Buisson N., Leonard H., Bailey M.E.S., Schanen N.C., Zappella M. (2010). Rett syndrome: Revised diagnostic criteria and nomenclature. Ann. Neurol..

[2] Rett A. (1966). On a unusual brain atrophy syndrome in hyperammonemia in childhood. Wien. Med. Wochenschr..

[3] Smeets E.E., Townend G.S., Curfs L.M.G. (2019). Rett syndrome and developmental regression. Neurosci. Biobehav. Rev..

[4] Amir R.E., Van den Veyver I.B., Wan M., Tran C.Q., Francke U., Zoghbi H.Y. (1999). Rett syndrome is caused by mutations in X-linked MECP2, encoding methyl-CpG-binding protein 2. Nat. Genet..

[5] Bienvenu T., Carrié A., de Roux N., Vinet M.-C., Jonveaux P., Couvert P., Chelly J. (2000). MECP2 mutations account for most cases of typical forms of Rett syndrome. Hum. Mol. Genet..

[6] Chahrour M., Zoghbi H.Y. (2007). The story of Rett syndrome: From clinic to neurobiology. Neuron.

[7] Masuyama T., Matsuo M., Jing J.J., Tabara Y., Kitsuki K., Yamagata H., Kondo I. (2005). Classic Rett syndrome in a boy with R133C mutation of MECP2. Brain Dev..

[8] Reichow B., George-Puskar A., Lutz T., Smith I.C., Volkmar F.R. (2015). Brief Report: Systematic Review of Rett Syndrome in Males. J. Autism Dev. Disord..

[9] Hagberg B., Goutières F., Hanefeld F., Rett A., Wilson J. (1985). Rett syndrome: Criteria for inclusion and exclusion. Brain Dev..

[10] Naidu S., Murphy M., Moser H.W., Rett A. (1986). Rett syndrome—Natural history in 70 cases. Am. J. Med. Genet. Suppl..

[11] Hagberg B. (2002). Clinical manifestations and stages of rett syndrome. Ment. Retard. Dev. Disabil. Res. Rev..

[12] Leonard H., Cobb S., Downs J. (2017). Clinical and biological progress over 50 years in Rett syndrome. Nat. Rev. Neurol..

[13] Downs J., Rodger J., Li C., Tan X., Hu N., Wong K., de Klerk N., Leonard H. (2018). Environmental enrichment intervention for Rett syndrome: An individually randomised stepped wedge trial. Orphanet J. Rare Dis..

[14] Singh J., Lanzarini E., Santosh P. (2020). Autonomic dysfunction and sudden death in patients with Rett syndrome: A systematic review. J. Psychiatry Neurosci..

[15] Singh J., Santosh P. (2018). Key issues in Rett syndrome: Emotional, behavioural and autonomic dysregulation (EBAD)—A target for clinical trials. Orphanet J. Rare Dis..

[16] Gomathi M., Padmapriya S., Balachandar V. (2020). Drug Studies on Rett Syndrome: From Bench to Bedside. J. Autism Dev. Disord..

[17] Katz D.M., Bird A., Coenraads M., Gray S.J., Menon D.U., Philpot B.D., Tarquinio D.C. (2016). Rett Syndrome: Crossing the Threshold to Clinical Translation. Trends Neurosci..

[18] Samaco R.C., Neul J.L. (2011). Complexities of Rett syndrome and MeCP2. J. Neurosci..

[19] Leonard H., Gold W., Samaco R., Sahin M., Benke T., Downs J. (2022). Improving clinical trial readiness to accelerate development of new therapeutics for Rett syndrome. Orphanet J. Rare Dis..

[20] Santosh P., Lievesley K., Fiori F., Singh J. (2017). Development of the Tailored Rett Intervention and Assessment Longitudinal (TRIAL) database and the Rett Evaluation of Symptoms and Treatments (REST) Questionnaire. BMJ Open.

[21] Downs J., Jacoby P., Leonard H., Epstein A., Murphy N., Davis E., Williams K. (2019). Psychometric properties of the Quality of Life Inventory-Disability (QI-Disability) measure. Qual. Life Res..

[22] Epstein A., Williams K., Reddihough D., Murphy N., Leonard H., Whitehouse A., Downs J. (2019). Content validation of the Quality of Life Inventory-Disability. Child Care Health Dev..

[23] Fitz Gerald P.M., Jankovic J., Percy A.K. (1990). Rett syndrome and associated movement disorders. Mov. Disord..

[24] Raspa M., Bann C.M., Gwaltney A., Benke T.A., Fu C., Glaze D.G., Haas R., Heydemann P., Jones M., Kaufmann W.E. (2020). A Psychometric Evaluation of the Motor-Behavioral Assessment Scale for Use as an Outcome Measure in Rett Syndrome Clinical Trials. Am. J. Intellect. Dev. Disabil..

[25] Rodocanachi Roidi M.L., Isaias I.U., Cozzi F., Grange F., Scotti F.M., Gestra V.F., Gandini A., Ripamonti E. (2019). A New Scale to Evaluate Motor Function in Rett Syndrome: Validation and Psychometric Properties. Pediatr. Neurol..

[26] Downs J., Stahlhut M., Wong K., Syhler B., Bisgaard A.M., Jacoby P., Leonard H. (2016). Validating the Rett Syndrome Gross Motor Scale. PLoS ONE.

[27] Busner J., Targum S.D. (2007). The clinical global impressions scale: Applying a research tool in clinical practice. Psychiatry.

[28] Neul J.L., Glaze D.G., Percy A.K., Feyma T., Beisang A., Dinh T., Suter B., Anagnostou E., Snape M., Horrigan J. (2015). Improving Treatment Trial Outcomes for Rett Syndrome: The Development of Rett-specific Anchors for the Clinical Global Impression Scale. J. Child Neurol..

[29] Mount R.H., Charman T., Hastings R.P., Reilly S., Cass H. (2002). The Rett Syndrome Behaviour Questionnaire (RSBQ): Refining the behavioural phenotype of Rett syndrome. J. Child Psychol. Psychiatry.

[30] Hou W., Bhattacharya U., Pradana W.A., Tarquinio D.C. (2020). Assessment of a Clinical Trial Metric for Rett Syndrome: Critical Analysis of the Rett Syndrome Behavioural Questionnaire. Pediatric Neurol..

[31] Hou W., Tarquinio D.C. (2020). Reply to Oberman et al. Pediatr. Neurol..

[32] Basch. E., Bennett. A.V. (2014). Patient-reported outcomes in clinical trials of rare diseases. J. Gen. Intern. Med..

[33] Bösch F., Zeltner N.A., Baumgartner M.R., Huemer M., Landolt M.A. (2022). Key patient-reported outcomes in children and adolescents with intoxication-type inborn errors of metabolism: An international Delphi-based consensus. Orphanet J. Rare Dis..

[34] Gualniera L., Singh J., Fiori F., Santosh P. (2021). Emotional Behavioural and Autonomic Dysregulation (EBAD) in Rett Syndrome—EDA and HRV monitoring using wearable sensor technology. J. Psychiatr. Res..

[35] Santosh P., Sagar-Ouriaghli I., Fiori F., Singh J. (2017). Using wearable sensor technology to manage EBAD (emotional, behavioural and autonomic dysregulation) in patients with complex neurodevelopment disorders. J. Psychopharmacol..

[36] Singh J., Santosh P., Malhotra S., Santosh P. (2016). Psychopharmacology of Neurodevelopmental Disorders in Children. Child and Adolescent Psychiatry Asian Perspectives.

[37] U.S. Department of Health and Human Services Food and Drug Administration (2019). Guidance for Industry: Patient-Reported Outcome Measures: Use in Medical Product Development to Support Labeling Claims Retrieved. https://www.fda.gov/regulatory-information/search-fda-guidance-documents/patient-reported-outcome-measures-use-medical-product-development-support-labeling-claims.

[38] Mokkink L.B., Prinsen C.A.C., Patrick D.L., Alonso J., Bouter L.M., de Vet H.C.W., Terwee C.B. (2019). COSMIN Study Design Checklist for Patient-Reported Outcome Measurement Instruments. https://www.cosmin.nl/wp-content/uploads/COSMIN-study-designing-checklist_final.pdf.

[39] Barnes K.V., Coughlin F.R., O’Leary H.M., Bruck N., Bazin G.A., Beinecke E.B., Walco A.C., Cantwell N.G., Kaufmann W.E. (2015). Anxiety-like behavior in Rett syndrome: Characteristics and assessment by anxiety scales. J. Neurodev. Disord..

[40] Kerr A.M., Nomura Y., Armstrong D., Anvret M., Belichenko P.V., Budden S., Cass H., Christodoulou J., Clarke A., Ellaway C. (2001). Guidelines for reporting clinical features in cases with MECP2 mutations. Brain Dev..

[41] Kaufmann W.E., Tierney E., Rohde C.A., Suarez-Pedraza M.C., Clarke M.A., Salorio C.F., Bibat G., Bukelis I., Naram D., Lanham D.C. (2012). Social impairments in Rett syndrome: Characteristics and relationship with clinical severity. J. Intellect. Disabil. Res..

[42] Rodríguez-Quiroga A., Flamarique I., Castro-Fornieles J., Lievesley K., Buitelaar J.K., Coghill D., Díaz-Caneja C.M., Dittmann R.W., Gupta A., Hoekstra P.J. (2020). Development and psychometric properties of the “Suicidality: Treatment Occurring in Paediatrics (STOP) Risk and Resilience Factors Scales” in adolescents. Eur. Child Adolesc. Psychiatry.

[43] Flamarique I., Santosh P., Zuddas A., Arango C., Purper-Ouakil D., Hoekstra P.J., Coghill D., Schulze U., Dittmann R.W., Buitelaar J.K. (2016). Development and psychometric properties of the Suicidality: Treatment Occurring in Paediatrics (STOP) Suicidality Assessment Scale (STOP-SAS) in children and adolescents. BMC Pediatrics.

[44] Santosh P., Singh J., Adams L., Mastroianni M., Heaney N., Lievesley K., Sagar-Ouriaghli I., Allibrio G., Appleton R., Davidović N. (2020). Validation of the Transition Readiness and Appropriateness Measure (TRAM) for the Managing the Link and Strengthening Transition from Child to Adult Mental Healthcare in Europe (MILESTONE) study. BMJ Open.

[45] Santosh P., Gringras P., Baird G., Fiori F., Sala R. (2015). Development and psychometric properties of the parent version of the Profile of Neuropsychiatric Symptoms (PONS) in children and adolescents. BMC Pediatrics.

[46] Hays R.D., Morales L.P., Reise S.P. (2000). Item response theory and health outcomes measurement in the 21st century. Med. Care.

[47] Nunnally J.C. (1978). Psychometric Theory.

[48] Oberman L.M., Downs J., Cianfaglione R., Leonard H., Kaufmann W.E. (2020). Assessment of a Clinical Trial Metric for Rett Syndrome: Critical Analysis of the Rett Syndrome Behaviour Questionnaire. Pediatric Neurol..

