# Supplementary material for: Development and Psychometric Properties of the Multi-System Profile of Symptoms Scale in Patients with Rett Syndrome
Source: J Clin Med. 2022 Aug 30;11(17):5094. doi: 10.3390/jcm11175094 (PMC9457440; doi:10.3390/jcm11175094)
Supplement: Supplementary file 1 [file jcm-11-05094-s001.zip › jcm-1847015-supplementary.pdf]

Supplementary Tables S1 (A and B)

A: Multi-System Profile of Symptoms Scale - Marginal fits (X^2) and Standardized Local Dependence X^2 Statistics

**Mental Health Problems**	X^2	1	2	3	4	5	6	
Aggression	0.2							
Self-Injury	0.4	1.7						
Screaming	0.5	1.1	1					
Fears	0.4	2.6	-0.1	2.1				
Agitation	1.1	2.5	0.1	2.5	3.2			
Panic Attacks	1	0.1	0.5	2.2	0.7	3.5		
Low Mood	0.2	2.1	-0.1	1	2.6	2	0.2	
**Autonomic Problems**	X^2	1	2	3	4	5	6	7
Temperature Changes	0.8							
Sweating	0.4	2.5						
Pupillary Changes	0.9	1.1	2.8					
Urination	0.1	-0.1	2	2.4				
Cold Limbs	0	0.8	0.5	2.4	3.7			
Breath-Holding During Sleep	0	1.6	-1.3	2.8	0.1	0.9		
Shallow Breathing	0	0.6	0.7	1.4	-0.2	-0.1	1.5	
Diarrhoea	0	0.9	1.1	0.5	1.1	-0.7	0.1	2.8
**Cardiac Problems**	X^2	1	2	3	4			
Irregular Heartbeat	0.5							
Fainting	0.7	----						
Sudden Changes in Heart Rate	1.1	5.7	6.2					
Postural Change	0.1	3.1	1.2	0.1				
Rapid Heartbeat	0.1	0.8	1.2	0.6	-0.4			
**Problems in Communication**	X^2	1	2	3				
Following Commands	0.1							
Understanding Words	0.1	0.2						
Non-Verbal Communication	0.2	-0.2	1.4					
Vocalisation	0.2	1.2	2.2	1				
**Problems in Social Behaviour**	X^2	1	2	3				
Being Touched or Held	0.3							
Repetitive Behaviour	1.3	1.8						
Routines	1.7	3.6	4.6					
Eye Contact	0.9	3.6	2.3	2.2				
**Problems in Engagement**	X^2	1	2	3	4	5		
Disengagement	0.2							

Lethargy	0.1	2.7						
Alertness	0	-1.4	-1.6					
Energy Level	0	-1.6	-1	0.7				
Drowsiness	0.1	2	1.1	0.2	3.5			
Dry Mouth	0.1	2.1	0.1	0.1	0.9	0.3		
**Gastrointestinal Problems**	X^2	1	2	3	4	5		
Abdominal Pain	0.2							
Pain After Meal	0.4	0.3						
Abdominal Bloating	0.3	-0.6	1					
Constipation	0.3	1.9	3.3	0.8				
Acid Reflux	0.1	1.2	0.7	1.4	2.1			
Toileting	0.1	0.4	1.1	-0.2	2.1	0.8		
**Problems in Motor Skills**	X^2	1	2	3	4	5		
Gross Motor Skills	0.1							
Fine Motor Skills	0.1	-0.1						
Clumsiness	0	-0.3	0					
Gait and Balance	0	2.5	-0.8	0.1				
Stereotypic Hand Movements	0	2.6	1.8	1	-0.2			
Writhing Limb Movements	0	-0.6	-0.6	0.2	0	1.5		
**Neurological Problems**	X^2	1	2	3	4	5		
Muscle Spasms	0.4							
Muscle Stiffness	0.3	0.6						
Abnormal Muscle Movements	0.1	-0.8	0.8					
Tremors	0.1	-0.3	-0.5	4.3				
Fasciculations	0.1	-0.1	-0.2	1.4	0.1			
Seizures	0.1	0.9	-0.1	1.2	-0.6	-1.5		
**Orofacial Problems**	X^2	1	2	3				
Chewing	0.8							
Swallowing	0.5	1.6						
Tongue Mobility	0.4	2.9	2.1					
Mouth Closure	0.1	2.7	2.3	2.3				
**Respiratory Problems**	X^2	1	2	3	4	5	6	7
Over-breathing	0.5							
Air Swallowing	0.5	2.9						
Breath-Holding When Awake	0.3	2.9	1.2					
Hyperventilation	0.5	2.3	1	0.9				
Air-Puffing	0.2	3.6	2.8	0.8	3.2			
Gasping	0	2.3	1	0	2.5	1.5		
Apnoea	0.1	2.1	2.7	1.9	1.5	0.7	0.3	

Cyanosis	0	3.3	2.6	0.8	1.2	2.1	1.9	7.4
**Sleep Problems**	X^2	1	2	3	4			
Nightmares and Night Terrors	0.1							
Night-Sweats	0.3	0.5						
Morning Wakefulness	0	-0.7	2.7					
Clamminess	0	0.6	0.9	0.8				
Insomnia	0	0.9	0.8	0	-0.7			

Abbreviations: X^2 (Chi-square)

Notes:
A Graded Parameter Response Model was run
Empty squares reflect those items that did not have anyone scoring at this level

B: Multi-System Profile of Symptoms Scale Supplement - Marginal fits (X^2) and Standardized Local Dependence X^2 Statistics

**Sensory Problems**	X^2	1	2	
Olfactory Function	0.2			
Auditory Function	0.2	4.5		
Visual Function	0.3	4.1	2	
**Immune Dysfunction and Infection**	X^2	1	2	3
Infections	1.3			
Respiratory Infections	0.5	1.2		
Urinary Tract Infections	0.5	3.5	2.5	
Food Intolerance	0.3	6	0.1	1.9
**Endocrine Problems**	X^2	1	2	3
Puberty	0.1			
Menstruation	0	2.3		
Growth	0.1			
Hormonal Problems	0.2	1.7	-0.5	
**Skeletal Problems**	X^2	1	2	
Scoliosis	0.2			
Fractures And Osteopenia	0	0.5		
Joint Problems	0.2	2.4	-0.7	
**Dermatological Problems**	X^2	1	2	3
Skin Rashes	0.7			
Skin Texture	1	1.4		
Skin Discoloration	0.6	1.5	0.1	
Other Skin Problems	0.3		1.5	1.4

Abbreviations: X^2 (Chi-square)

Notes:
A Graded Parameter Response Model was run
Empty squares reflect those items that did not have anyone scoring at this level

Supplementary Tables S2 (A and B)

A: Multi-System Profile of Symptoms Scale - Theta Scores

**Mental Health Problems**	Theta (θ)														
	-2.8	-2.4	-2	-1.6	-1.2	-0.8	-0.4	0	0.4	0.8	1.2	1.6	2	2.4	2.8
Aggression	0	0	0.01	0.03	0.09	0.25	0.64	1.35	2.05	2.32	2.29	2.07	1.44	0.71	0.28
Self-Injury	0	0	0.01	0.02	0.06	0.19	0.53	1.24	2.1	2.49	2.44	1.96	1.09	0.45	0.16
Screaming	0.01	0.02	0.05	0.12	0.31	0.72	1.33	1.83	1.98	1.96	1.93	1.66	1.07	0.52	0.22
Fears	0	0.01	0.02	0.06	0.15	0.36	0.77	1.34	1.73	1.84	1.82	1.74	1.39	0.82	0.39
Agitation	0.01	0.04	0.15	0.48	1.34	2.55	3.07	3.09	3.01	2.81	2.65	1.71	0.68	0.22	0.06
Panic Attacks	0.01	0.03	0.07	0.16	0.35	0.68	1.08	1.38	1.48	1.48	1.46	1.38	1.13	0.73	0.39
Low Mood	0.03	0.06	0.11	0.21	0.38	0.62	0.85	1	1.05	1.07	1.06	1	0.84	0.6	0.37
Test Information:	1.06	1.16	1.42	2.09	3.69	6.36	9.28	12.22	14.41	14.96	14.64	12.51	8.64	5.05	2.87
Expected s.e.:	0.97	0.93	0.84	0.69	0.52	0.4	0.33	0.29	0.26	0.26	0.26	0.28	0.34	0.44	0.59
**Autonomic Problems**	-2.8	-2.4	-2	-1.6	-1.2	-0.8	-0.4	0	0.4	0.8	1.2	1.6	2	2.4	2.8
Temperature Changes	0	0.02	0.05	0.15	0.43	1.07	1.97	2.43	2.55	2.56	2.54	2.44	1.83	0.92	0.35
Sweating	0	0.01	0.01	0.04	0.11	0.28	0.66	1.29	1.81	1.99	2.06	2.03	1.92	1.61	0.99
Pupillary Changes	0	0.01	0.02	0.06	0.16	0.42	0.99	1.75	2.18	2.27	2.25	2.16	1.66	0.89	0.37
Urination	0.01	0.02	0.04	0.07	0.14	0.24	0.41	0.6	0.77	0.86	0.9	0.92	0.92	0.88	0.75
Cold Limbs	0.26	0.34	0.42	0.47	0.5	0.51	0.51	0.5	0.47	0.42	0.35	0.27	0.19	0.13	0.08
Breath-Holding During Sleep	0.02	0.04	0.06	0.09	0.13	0.19	0.26	0.33	0.39	0.43	0.45	0.46	0.46	0.45	0.45
Shallow Breathing	0.05	0.07	0.11	0.14	0.19	0.24	0.28	0.31	0.34	0.35	0.35	0.35	0.34	0.32	0.28
Diarrhoea	0.05	0.06	0.08	0.1	0.12	0.14	0.16	0.18	0.19	0.2	0.2	0.2	0.21	0.21	0.21
Test Information:	1.4	1.56	1.78	2.12	2.77	4.1	6.24	8.38	9.69	10.08	10.11	9.83	8.53	6.41	4.48
Expected s.e.:	0.84	0.8	0.75	0.69	0.6	0.49	0.4	0.35	0.32	0.32	0.31	0.32	0.34	0.4	0.47
**Cardiac Problems**	-2.8	-2.4	-2	-1.6	-1.2	-0.8	-0.4	0	0.4	0.8	1.2	1.6	2	2.4	2.8
Irregular Heartbeat	0	0	0	0	0	0	0.02	0.17	1.03	4.34	7.01	7.28	5.72	1.74	0.3
Fainting	0	0	0	0	0	0	0	0.02	0.09	0.45	1.85	4.42	5.28	4.42	1.85
Sudden Changes in Heart Rate	0	0	0	0	0	0.01	0.03	0.22	1.33	4.93	7.09	7.01	5.46	1.68	0.29
Postural Change	0	0	0	0	0.01	0.02	0.07	0.19	0.51	1.13	1.88	2.24	2.09	1.47	0.73
Rapid Heartbeat	0.03	0.06	0.11	0.22	0.38	0.61	0.83	0.97	1.02	1.03	1.01	0.98	0.91	0.75	0.53
Test Information:	1.03	1.06	1.12	1.22	1.39	1.64	1.96	2.57	4.97	12.88	19.85	22.92	20.46	11.06	4.7
Expected s.e.:	0.99	0.97	0.95	0.91	0.85	0.78	0.71	0.62	0.45	0.28	0.22	0.21	0.22	0.3	0.46
**Problems in Communication**	-2.8	-2.4	-2	-1.6	-1.2	-0.8	-0.4	0	0.4	0.8	1.2	1.6	2	2.4	2.8
Following Commands	0.12	0.5	1.71	3.5	3.93	4.28	4.28	3.89	2.23	0.71	0.18	0.04	0.01	0	0
Understanding Words	0.08	0.23	0.66	1.51	2.35	2.62	2.6	2.55	2.47	1.77	0.84	0.31	0.1	0.03	0.01

Non-Verbal Communication	0.25	0.47	0.79	1.09	1.26	1.31	1.29	1.18	0.91	0.58	0.31	0.15	0.07	0.03	0.01
Vocalisation	0.49	0.69	0.84	0.9	0.9	0.82	0.67	0.47	0.29	0.16	0.09	0.05	0.02	0.01	0.01
Test Information:	1.93	2.89	5	8	9.43	10.03	9.84	9.08	6.89	4.22	2.42	1.55	1.21	1.08	1.03
Expected s.e.:	0.72	0.59	0.45	0.35	0.33	0.32	0.32	0.33	0.38	0.49	0.64	0.8	0.91	0.96	0.98
**Problems in Social Behaviour**	-2.8	-2.4	-2	-1.6	-1.2	-0.8	-0.4	0	0.4	0.8	1.2	1.6	2	2.4	2.8
Being Touched or Held	0.01	0.02	0.04	0.08	0.14	0.24	0.38	0.55	0.69	0.77	0.81	0.81	0.79	0.73	0.6
Repetitive Behaviour	0.01	0.02	0.06	0.17	0.45	1	1.72	2.13	2.18	1.93	1.29	0.63	0.25	0.09	0.03
Routines	0	0	0	0	0.02	0.31	3.91	13.53	13.25	13.52	3.71	0.29	0.02	0	0
Eye Contact	0.04	0.08	0.14	0.25	0.41	0.6	0.77	0.86	0.89	0.88	0.82	0.68	0.49	0.31	0.18
Test Information:	1.06	1.12	1.25	1.5	2.02	3.16	7.78	18.08	18.01	18.1	7.63	3.41	2.56	2.13	1.82
Expected s.e.:	0.97	0.94	0.9	0.82	0.7	0.56	0.36	0.24	0.24	0.24	0.36	0.54	0.63	0.68	0.74
**Problems in Engagement**	-2.8	-2.4	-2	-1.6	-1.2	-0.8	-0.4	0	0.4	0.8	1.2	1.6	2	2.4	2.8
Disengagement	0	0.01	0.03	0.11	0.37	1.03	2.13	2.79	2.96	2.98	2.87	2.41	1.33	0.5	0.16
Lethargy	0	0	0.02	0.09	0.46	2.06	4.9	5.6	5.67	5.53	5.14	2.7	0.66	0.13	0.02
Alertness	0.03	0.05	0.11	0.22	0.4	0.67	0.93	1.1	1.15	1.15	1.1	0.96	0.7	0.43	0.24
Energy Level	0.02	0.04	0.08	0.16	0.31	0.55	0.85	1.08	1.19	1.19	1.11	0.9	0.61	0.35	0.18
Drowsiness	0.05	0.09	0.15	0.24	0.37	0.52	0.64	0.71	0.72	0.68	0.58	0.44	0.3	0.19	0.11
Dry Mouth	0.05	0.06	0.08	0.11	0.14	0.17	0.2	0.22	0.24	0.25	0.26	0.26	0.26	0.26	0.26
Test Information:	1.14	1.25	1.47	1.93	3.05	6	10.64	12.5	12.93	12.78	12.05	8.67	4.86	2.86	1.97
Expected s.e.:	0.94	0.89	0.83	0.72	0.57	0.41	0.31	0.28	0.28	0.28	0.29	0.34	0.45	0.59	0.71
**Gastrointestinal Problems**	-2.8	-2.4	-2	-1.6	-1.2	-0.8	-0.4	0	0.4	0.8	1.2	1.6	2	2.4	2.8
Abdominal Pain	0	0	0.02	0.11	0.57	2.33	4.95	5.29	5.32	5.45	4.6	1.85	0.42	0.08	0.02
Pain After Meal	0	0	0.01	0.03	0.16	0.74	2.64	4.68	5.03	5.15	4.95	3.28	1.04	0.23	0.05
Abdominal Bloating	0.03	0.08	0.2	0.5	1.05	1.65	1.92	2.02	2.06	1.99	1.62	0.97	0.44	0.18	0.07
Constipation	0.06	0.15	0.34	0.69	1.14	1.48	1.61	1.64	1.61	1.42	1.01	0.56	0.27	0.11	0.05
Acid Reflux	0.05	0.09	0.15	0.24	0.36	0.49	0.6	0.67	0.7	0.7	0.69	0.64	0.55	0.42	0.29
Toileting	0.22	0.29	0.35	0.41	0.44	0.46	0.46	0.45	0.42	0.38	0.31	0.24	0.17	0.12	0.08
Test Information:	1.36	1.6	2.07	2.97	4.72	8.15	13.18	15.76	16.15	16.1	14.18	8.55	3.89	2.15	1.55
Expected s.e.:	0.86	0.79	0.7	0.58	0.46	0.35	0.28	0.25	0.25	0.25	0.27	0.34	0.51	0.68	0.8
**Problems in Motor Skills**	-2.8	-2.4	-2	-1.6	-1.2	-0.8	-0.4	0	0.4	0.8	1.2	1.6	2	2.4	2.8
Gross Motor Skills	0.05	0.29	1.44	4.49	5.99	5.51	5.76	4.25	1.31	0.26	0.05	0.01	0	0	0
Fine Motor Skills	0.06	0.35	1.88	5.59	6.75	6.44	4.73	1.35	0.24	0.04	0.01	0	0	0	0
Clumsiness	0.28	0.72	1.48	2.14	2.36	2.34	2.02	1.26	0.56	0.21	0.07	0.03	0.01	0	0
Gait And Balance	0.25	0.51	0.89	1.25	1.43	1.47	1.46	1.34	1.04	0.64	0.33	0.15	0.07	0.03	0.01
Stereotypic Hand Movements	0.37	0.38	0.39	0.38	0.38	0.36	0.32	0.28	0.22	0.17	0.12	0.09	0.06	0.04	0.03
Writhing Limb Movements	0.09	0.13	0.19	0.26	0.33	0.39	0.44	0.46	0.45	0.42	0.37	0.29	0.22	0.16	0.11

Test Information:	2.1	3.38	7.27	15.11	18.23	17.52	15.72	9.93	4.83	2.74	1.95	1.57	1.36	1.23	1.15
Expected s.e.:	0.69	0.54	0.37	0.26	0.23	0.24	0.25	0.32	0.46	0.6	0.72	0.8	0.86	0.9	0.93
**Neurological Problems**	-2.8	-2.4	-2	-1.6	-1.2	-0.8	-0.4	0	0.4	0.8	1.2	1.6	2	2.4	2.8
Muscle Spasms	0	0.01	0.03	0.15	0.73	2.7	4.82	5.26	5.2	4.66	4.34	1.92	0.46	0.09	0.02
Muscle Stiffness	0.05	0.14	0.38	0.95	1.77	2.23	2.37	2.45	2.36	2.23	2	1.25	0.55	0.2	0.07
Abnormal Muscle Movements	0.02	0.05	0.15	0.4	0.94	1.67	2.11	2.22	2.21	2.13	2.03	1.7	1	0.44	0.17
Tremors	0.02	0.06	0.14	0.32	0.68	1.16	1.5	1.6	1.69	1.72	1.64	1.38	0.9	0.46	0.21
Fasciculations	0.04	0.07	0.11	0.18	0.26	0.36	0.45	0.52	0.56	0.57	0.58	0.57	0.56	0.54	0.48
Seizures	0.07	0.11	0.16	0.22	0.28	0.34	0.39	0.41	0.43	0.43	0.43	0.42	0.41	0.38	0.34
Test Information:	1.21	1.43	1.98	3.23	5.67	9.47	12.64	13.48	13.44	12.75	12.02	8.25	4.88	3.11	2.28
Expected s.e.:	0.91	0.84	0.71	0.56	0.42	0.33	0.28	0.27	0.27	0.28	0.29	0.35	0.45	0.57	0.66
**Orofacial Problems**	-2.8	-2.4	-2	-1.6	-1.2	-0.8	-0.4	0	0.4	0.8	1.2	1.6	2	2.4	2.8
Chewing	0	0	0	0.01	0.17	2.05	10.34	10.24	12.08	11.34	2.29	0.19	0.01	0	0
Swallowing	0.01	0.02	0.05	0.16	0.46	1.14	2.05	2.45	2.61	2.63	2.12	1.17	0.46	0.16	0.05
Tongue Mobility	0	0.01	0.02	0.07	0.2	0.55	1.29	2.15	2.53	2.57	2.41	1.75	0.87	0.33	0.11
Mouth Closure	0.03	0.06	0.1	0.17	0.27	0.4	0.52	0.62	0.67	0.68	0.65	0.58	0.46	0.33	0.22
Test Information:	1.04	1.08	1.18	1.41	2.09	5.13	15.2	16.45	18.88	18.21	8.47	4.69	2.8	1.82	1.38
Expected s.e.:	0.98	0.96	0.92	0.84	0.69	0.44	0.26	0.25	0.23	0.23	0.34	0.46	0.6	0.74	0.85
**Respiratory Problems**	-2.8	-2.4	-2	-1.6	-1.2	-0.8	-0.4	0	0.4	0.8	1.2	1.6	2	2.4	2.8
Over-breathing	0	0	0.02	0.07	0.25	0.87	2.31	3.55	3.81	3.73	2.97	1.39	0.43	0.12	0.03
Air Swallowing	0	0.01	0.03	0.11	0.34	1	2.16	2.94	3.05	2.99	2.91	2.17	1.02	0.35	0.11
Breath-Holding When Awake	0.02	0.05	0.12	0.32	0.74	1.39	1.91	2.07	2.08	2.03	1.76	1.16	0.56	0.23	0.09
Hyperventilation	0.03	0.07	0.15	0.31	0.58	0.92	1.19	1.31	1.33	1.32	1.29	1.17	0.9	0.56	0.3
Air-Puffing	0.01	0.03	0.06	0.12	0.23	0.43	0.7	0.96	1.11	1.15	1.15	1.12	1.05	0.88	0.61
Gasping	0.03	0.05	0.08	0.14	0.23	0.36	0.5	0.63	0.7	0.73	0.74	0.72	0.67	0.57	0.44
Apnoea	0.01	0.01	0.02	0.04	0.08	0.13	0.21	0.33	0.46	0.57	0.64	0.67	0.69	0.69	0.68
Cyanosis	0.01	0.02	0.03	0.05	0.07	0.11	0.17	0.24	0.31	0.38	0.44	0.47	0.48	0.49	0.49
Test Information:	1.11	1.23	1.51	2.15	3.53	6.23	10.15	13.01	13.85	13.92	12.9	9.87	6.8	4.89	3.73
Expected s.e.:	0.95	0.9	0.81	0.68	0.53	0.4	0.31	0.28	0.27	0.27	0.28	0.32	0.38	0.45	0.52
**Sleep Problems**	-2.8	-2.4	-2	-1.6	-1.2	-0.8	-0.4	0	0.4	0.8	1.2	1.6	2	2.4	2.8
Nightmares and Night Terrors	0	0	0	0.01	0.03	0.1	0.28	0.73	1.5	2.13	2.33	2.38	2.37	2.23	1.65
Night-Sweats	0	0	0	0.01	0.04	0.11	0.3	0.73	1.44	2.01	2.2	2.21	2.19	2.04	1.47
Morning Wakefulness	0.03	0.05	0.08	0.13	0.21	0.32	0.43	0.53	0.59	0.62	0.64	0.64	0.63	0.6	0.53
Clamminess	0.06	0.09	0.14	0.22	0.31	0.4	0.48	0.52	0.55	0.56	0.56	0.55	0.53	0.49	0.41
Insomnia	0.05	0.08	0.13	0.19	0.26	0.34	0.41	0.45	0.48	0.48	0.48	0.47	0.45	0.4	0.34

Test Information:	1.14	1.23	1.36	1.56	1.85	2.26	2.89	3.96	5.55	6.81	7.21	7.26	7.18	6.77	5.4
Expected s.e.:	0.94	0.9	0.86	0.8	0.73	0.66	0.59	0.5	0.42	0.38	0.37	0.37	0.37	0.38	0.43

Abbreviations: *s.e.* (standard error)

Notes:
A Graded Parameter Response Model was run
Item Information Function Values for Group 1 at 15 Values of θ from -2.8 to 2.8

B: Multi-System Profile of Symptoms Scale Supplement - Theta Scores

	Theta (θ)														
**Sensory Problems**	-2.8	-2.4	-2	-1.6	-1.2	-0.8	-0.4	0	0.4	0.8	1.2	1.6	2	2.4	2.8
Olfactory Function	0	0	0	0	0	0.01	0.04	0.11	0.3	0.74	1.45	1.99	2.11	2.02	1.42
Auditory Function	0	0	0	0	0.01	0.03	0.1	0.36	1.17	2.62	3.31	3.54	3.6	2.95	1.45
Visual Function	0	0.01	0.01	0.03	0.06	0.13	0.26	0.49	0.79	1.04	1.16	1.21	1.21	1.14	0.94
Test Information:	1	1.01	1.01	1.03	1.07	1.17	1.4	1.96	3.25	5.4	6.93	7.73	7.92	7.11	4.81
Expected s.e.:	1	1	0.99	0.98	0.97	0.92	0.85	0.71	0.55	0.43	0.38	0.36	0.36	0.37	0.46
**Immune Dysfunction and Infection**	-2.8	-2.4	-2	-1.6	-1.2	-0.8	-0.4	0	0.4	0.8	1.2	1.6	2	2.4	2.8
Infections	0	0	0	0	0	0	0.02	0.17	1.21	5.36	7.78	7.82	7.11	2.99	0.5
Respiratory Infections	0	0	0	0	0	0	0.01	0.07	0.37	1.66	4.41	5.44	5.1	3.25	0.93
Urinary Tract Infections	0	0	0	0	0.01	0.02	0.06	0.18	0.48	1.09	1.81	2.12	2.17	2.12	1.82
Food Intolerance	0	0	0	0	0.01	0.03	0.09	0.27	0.69	1.45	2.15	2.38	2.24	1.62	0.82
Test Information:	1	1	1	1.01	1.02	1.06	1.19	1.69	3.75	10.56	17.15	18.76	17.62	10.98	5.08
Expected s.e.:	1	1	1	1	0.99	0.97	0.92	0.77	0.52	0.31	0.24	0.23	0.24	0.3	0.44
**Endocrine Problems**	-2.8	-2.4	-2	-1.6	-1.2	-0.8	-0.4	0	0.4	0.8	1.2	1.6	2	2.4	2.8
Puberty	0	0	0	0	0	0.01	0.02	0.08	0.3	0.95	2.27	3.27	3.44	2.98	1.65
Menstruation	0	0	0.01	0.02	0.03	0.06	0.12	0.23	0.4	0.62	0.84	0.96	0.96	0.84	0.63
Growth	0	0	0	0	0	0	0	0	0.01	0.16	1.84	10.28	11.71	10.38	2.3
Hormonal Problems	0	0	0	0	0	0	0.01	0.04	0.13	0.37	0.96	1.87	2.44	2.23	1.38
Test Information:	1	1	1.01	1.02	1.03	1.07	1.16	1.35	1.83	3.11	6.91	17.38	19.55	17.43	6.95
Expected s.e.:	1	1	1	0.99	0.98	0.97	0.93	0.86	0.74	0.57	0.38	0.24	0.23	0.24	0.38
**Skeletal Problems**	-2.8	-2.4	-2	-1.6	-1.2	-0.8	-0.4	0	0.4	0.8	1.2	1.6	2	2.4	2.8
Scoliosis	0.01	0.02	0.04	0.06	0.1	0.15	0.23	0.32	0.42	0.5	0.54	0.56	0.56	0.54	0.48
Fractures And Osteopenia	0	0	0.01	0.01	0.01	0.02	0.03	0.04	0.06	0.08	0.11	0.15	0.18	0.22	0.25
Joint Problems	0	0	0	0	0	0.01	0.06	0.53	3.65	8.97	9.23	8.92	3.45	0.49	0.06
Test Information:	1.02	1.03	1.04	1.07	1.11	1.18	1.32	1.89	5.13	10.55	10.89	10.63	5.19	2.25	1.79
Expected s.e.:	0.99	0.99	0.98	0.97	0.95	0.92	0.87	0.73	0.44	0.31	0.3	0.31	0.44	0.67	0.75
**Dermatological Problems**	-2.8	-2.4	-2	-1.6	-1.2	-0.8	-0.4	0	0.4	0.8	1.2	1.6	2	2.4	2.8
Skin Rashes	0	0	0	0	0	0	0.01	0.06	0.57	4.05	8.59	9.73	9.17	7.88	1.96
Skin Texture	0	0	0	0	0	0	0	0.02	0.42	8.23	19.76	20.49	13.47	0.91	0.04
Skin Discoloration	0	0	0	0	0	0	0.02	0.14	0.84	3.78	6.91	6.96	4.97	1.34	0.23
Other Skin Problems	0	0	0	0	0	0	0.01	0.04	0.14	0.43	1.15	2.23	2.78	2.71	2.11
Test Information:	1	1	1	1	1	1.01	1.04	1.26	2.96	17.49	37.41	40.41	31.4	13.84	5.33

Expected s.e.:	1	1	1	1	1	1	0.98	0.89	0.58	0.24	0.16	0.16	0.18	0.27	0.43

Abbreviations: *s.e.* (standard error)

Notes:
A Graded Parameter Response Model was run
Item Information Function Values for Group 1 at 15 Values of θ from -2.8 to 2.8

Supplementary Figure S1 (A and B)

A: Multi-System Profile of Symptoms Scale – Item Curve Characteristics

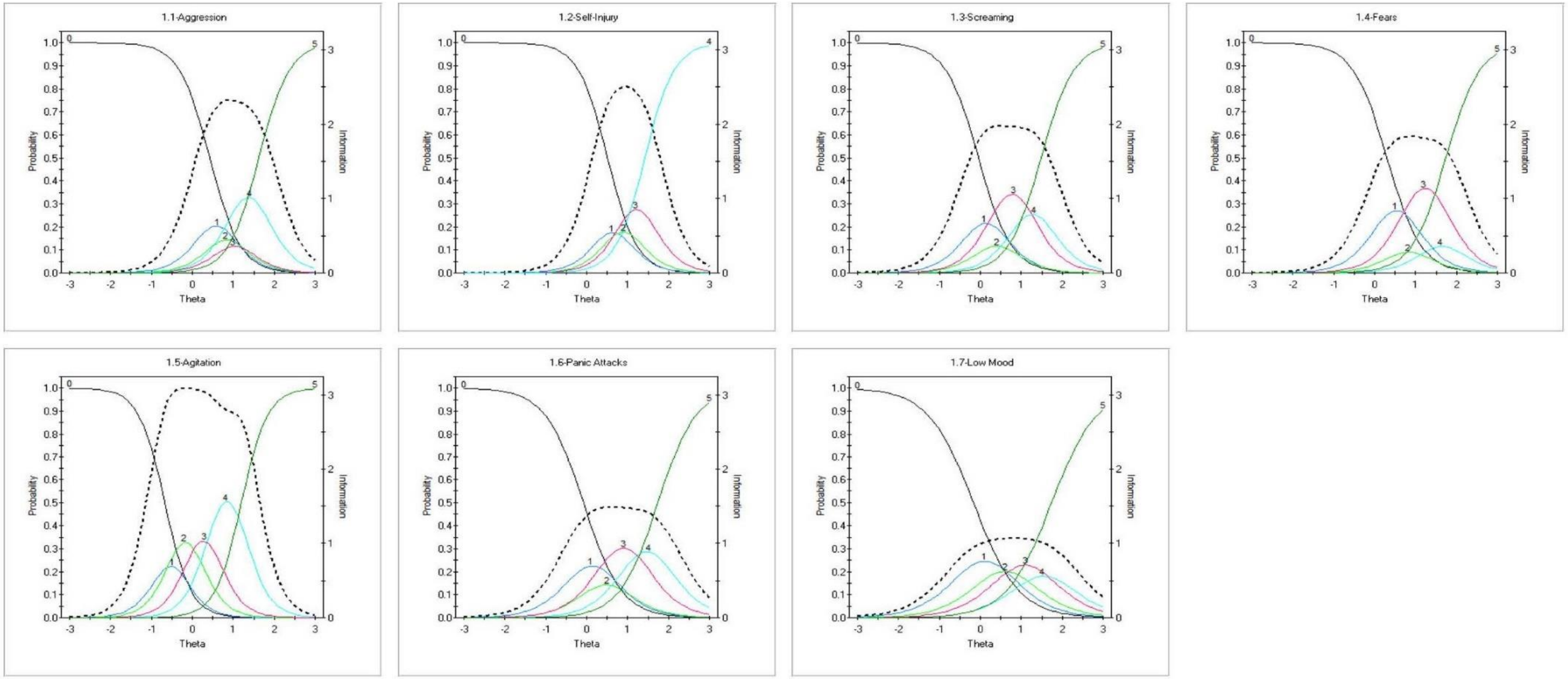

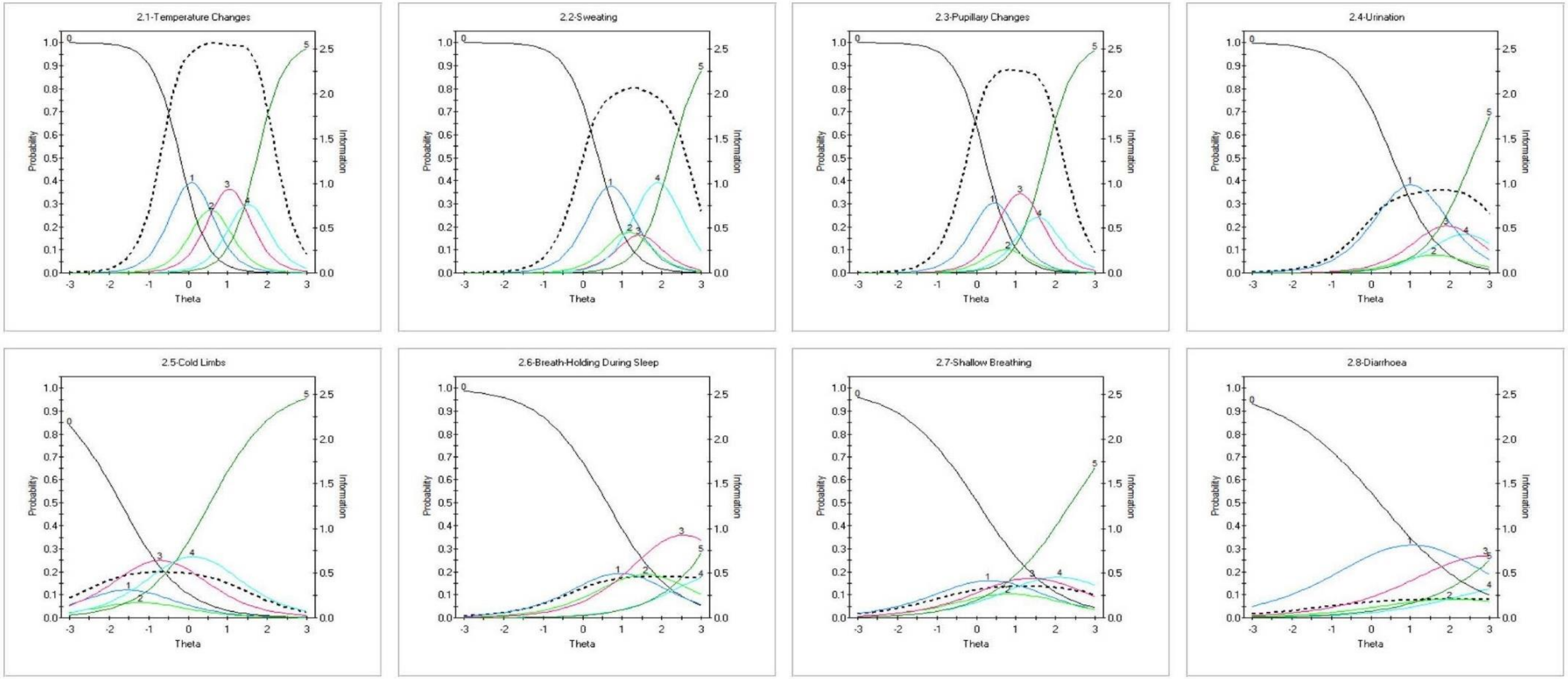
2.1-Temperature Changes
2.2-Sweating
2.3-Pupillary Changes
2.4-Urination
2.5-Cold Limbs
2.6-Breath-Holding During Sleep
2.7-Shallow Breathing
2.8-Diarrhoea
Probability
Information
Theta

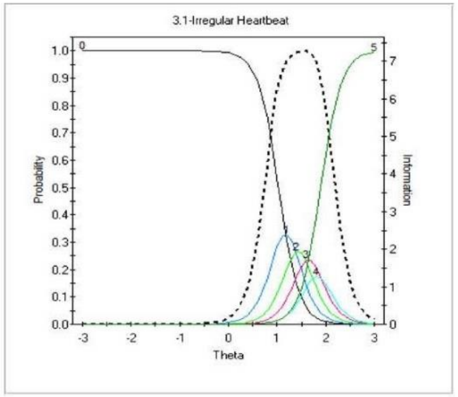
3.1-Irregular Heartbeat
Probability
Information
Theta

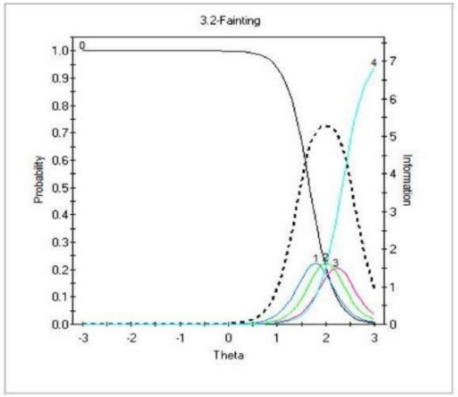
3.2-Fainting
Probability
Information
Theta

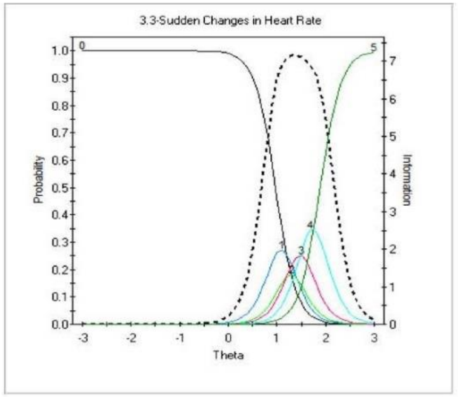
3.3-Sudden Changes in Heart Rate
Probability
Information
Theta

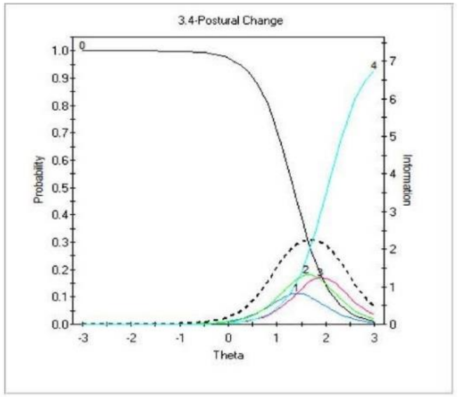
3.4-Postural Change
Probability
Information
Theta

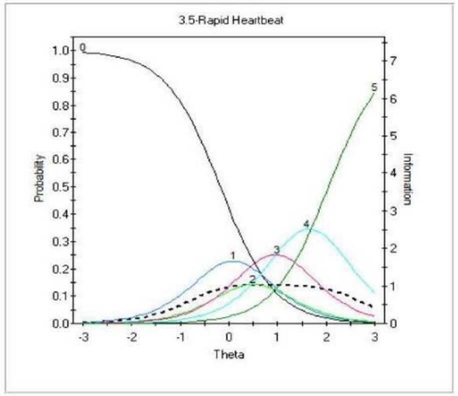
3.5-Rapid Heartbeat
Probability
Information
Theta

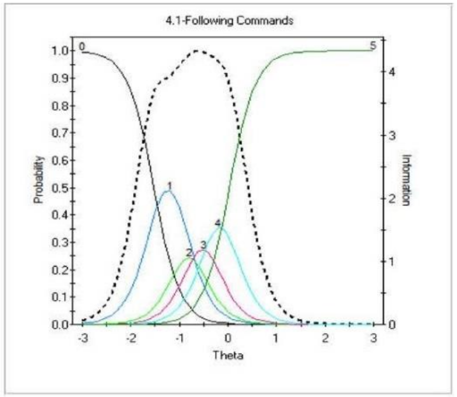
4.1-Following Commands
Probability
Information
Theta

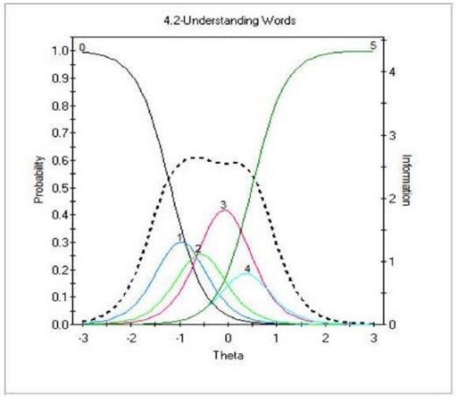
4.2-Understanding Words
Probability
Information
Theta

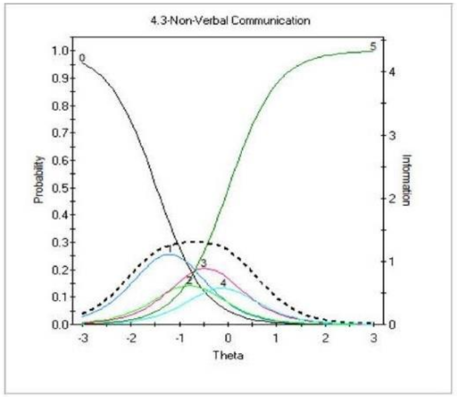
4.3-Non-Verbal Communication
Probability
Information
Theta

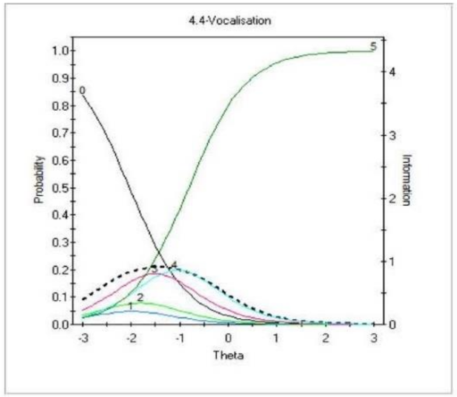
4.4-Vocalisation
Probability
Information
Theta

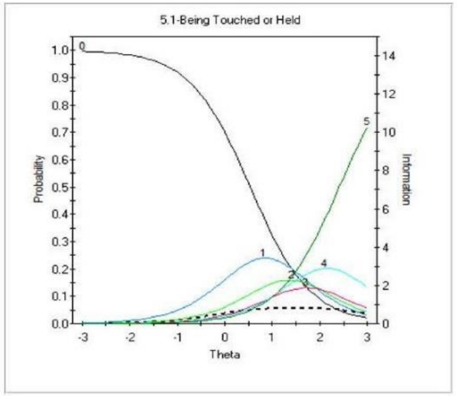
5.1-Being Touched or Held
Probability
Information
Theta

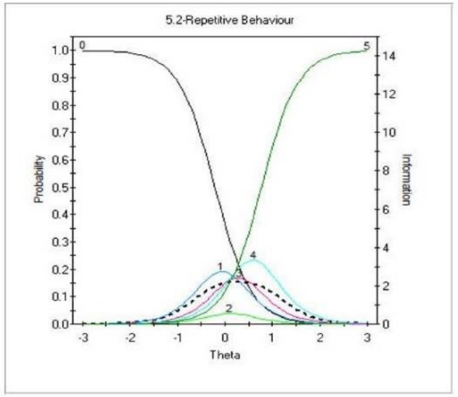
5.2-Repetitive Behaviour
Probability
Information
Theta

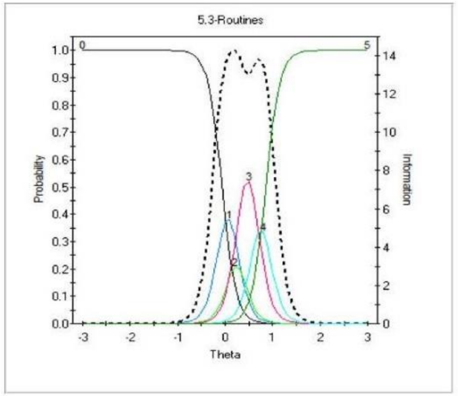
5.3-Routines
Probability
Information
Theta

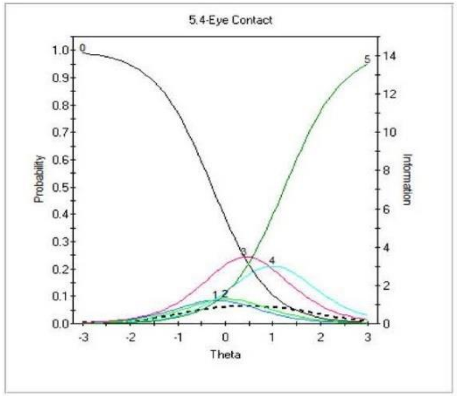
5.4-Eye Contact
Probability
Information
Theta

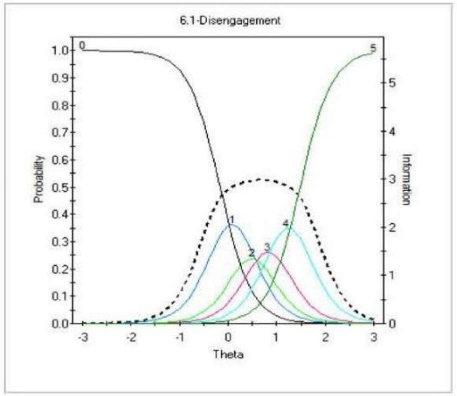
6.1-Disengagement
Probability
Information
Theta

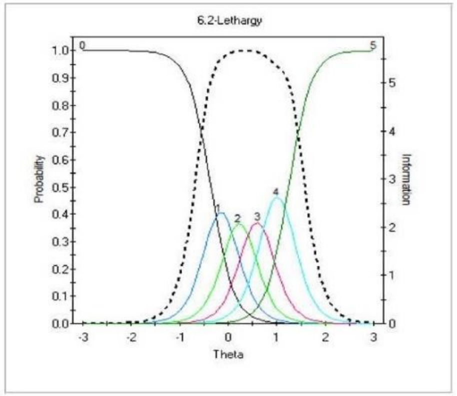
6.2-Lethargy
Probability
Information
Theta

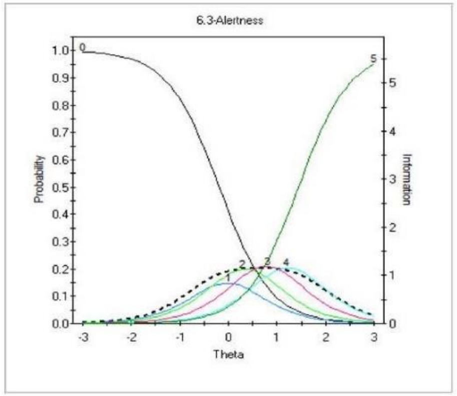
6.3-Alertness
Probability
Information
Theta

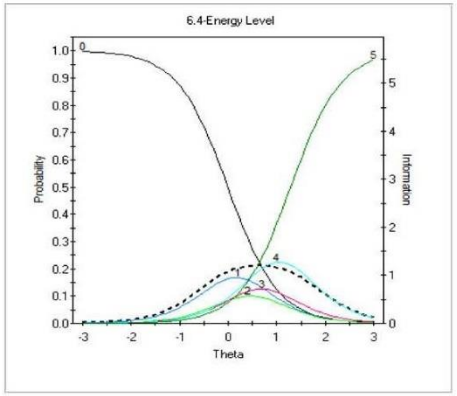
6.4-Energy Level
Probability
Information
Theta

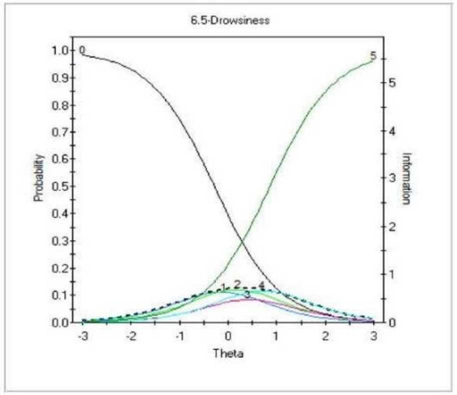
6.5-Drowsiness
Probability
Information
Theta

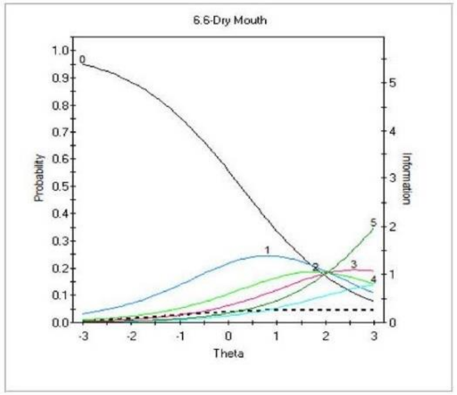
6.6-Dry Mouth
Probability
Information
Theta

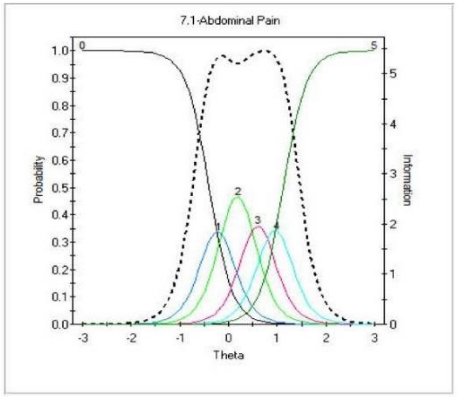
7.1-Abdominal Pain
Probability
Information
Theta

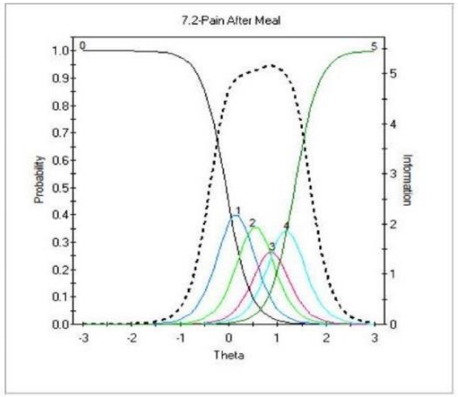
7.2-Pain After Meal
Probability
Information
Theta

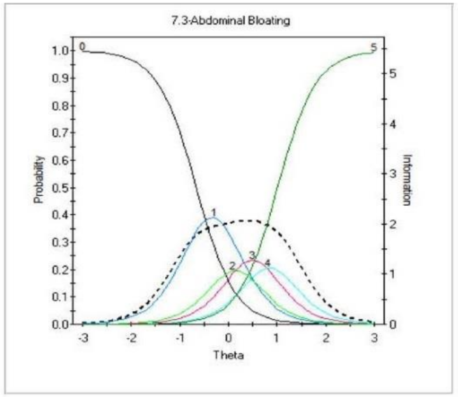
7.3-Abdominal Bloating
Probability
Information
Theta

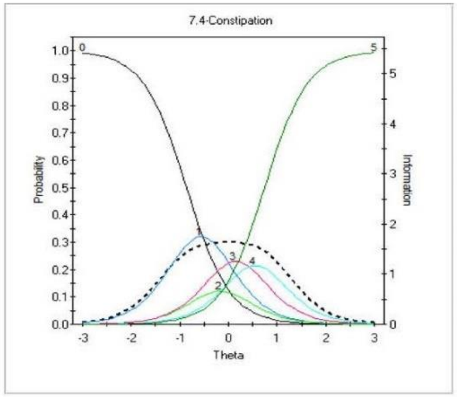
7.4-Constipation
Probability
Information
Theta

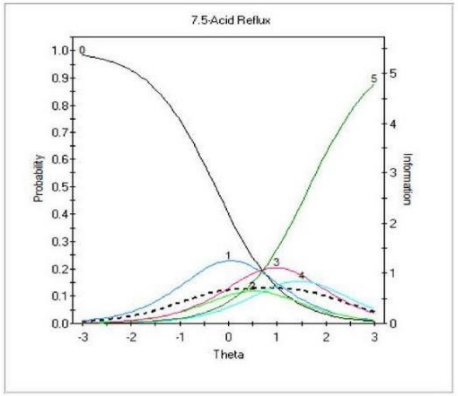
7.5-Acid Reflux
Probability
Information
Theta

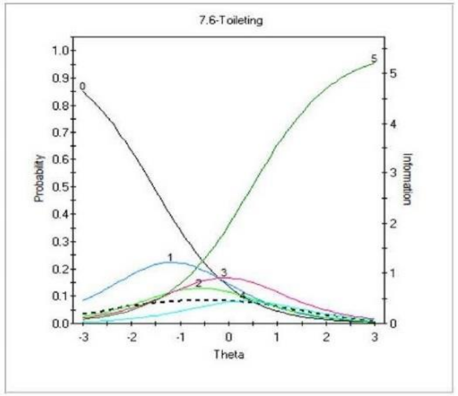
7.6-Toileting
Probability
Information
Theta

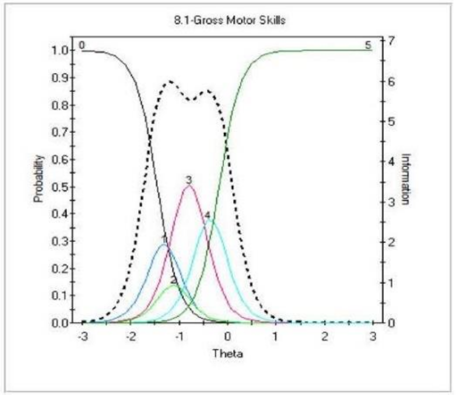
8.1-Gross Motor Skills
Probability
Information
Theta

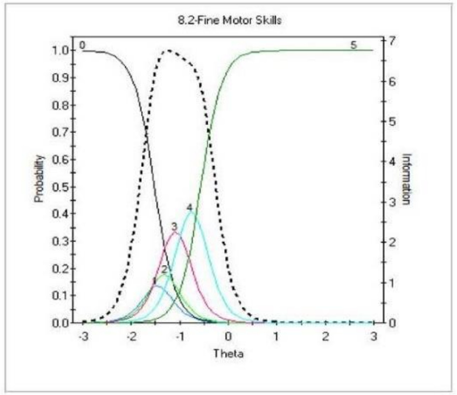
8.2-Fine Motor Skills
Probability
Information
Theta

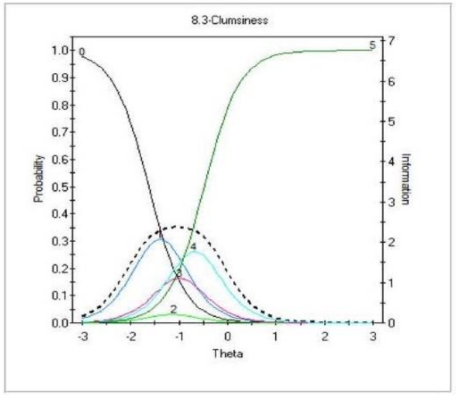
8.3-Clumsiness
Probability
Information
Theta

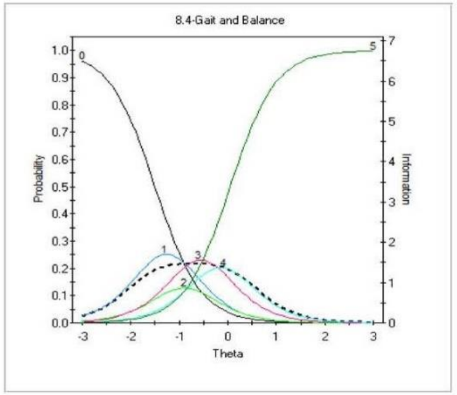
8.4-Gait and Balance
Probability
Information
Theta

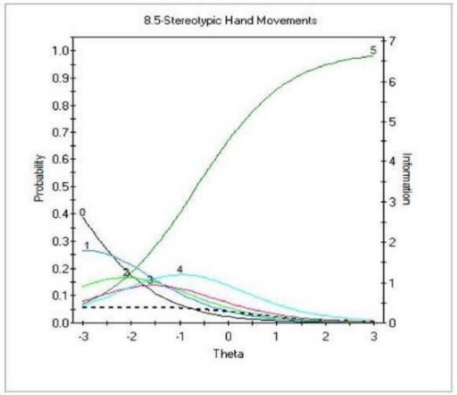
8.5-Stereotypic Hand Movements
Probability
Information
Theta

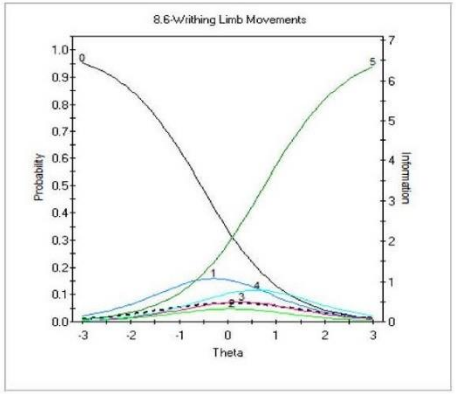
8.6-Writhing Limb Movements
Probability
Information
Theta

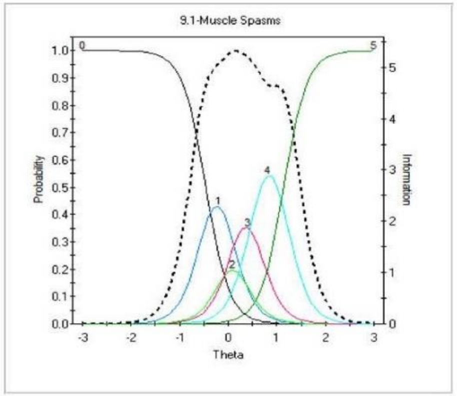
9.1-Muscle Spasms
Probability
Information
Theta

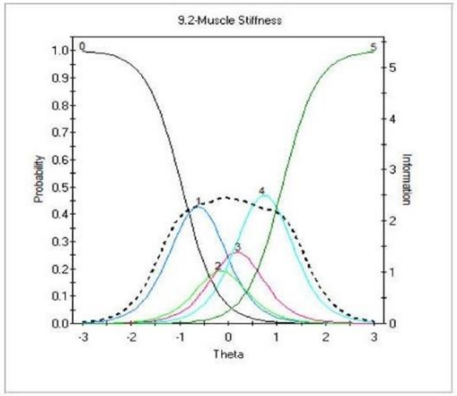
9.2-Muscle Stiffness
Probability
Information
Theta

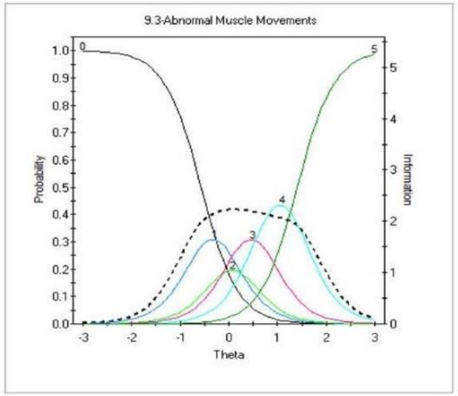
9.3-Abnormal Muscle Movements
Probability
Information
Theta

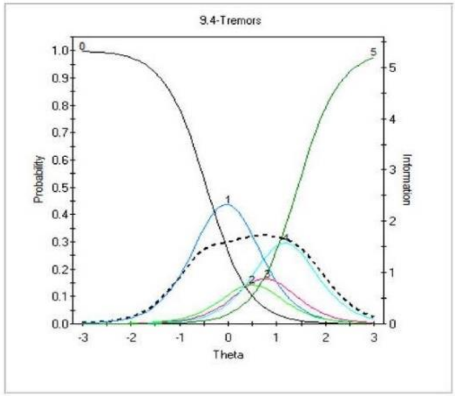
9.4-Tremors
Probability
Information
Theta

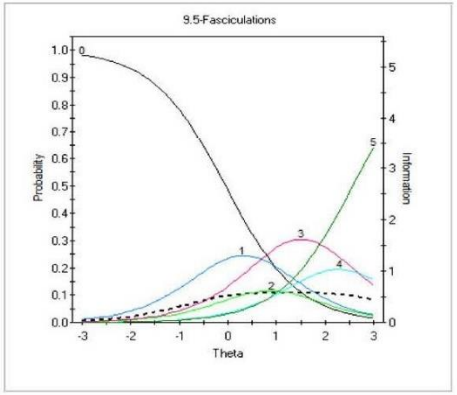
9.5-Fasciculations
Probability
Information
Theta

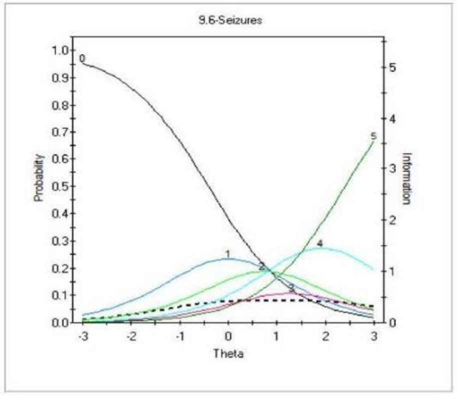
9.6-Seizures
Probability
Information
Theta

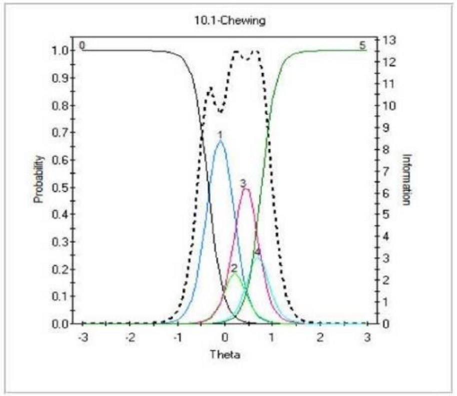
10.1-Chewing
Probability
Information
Theta

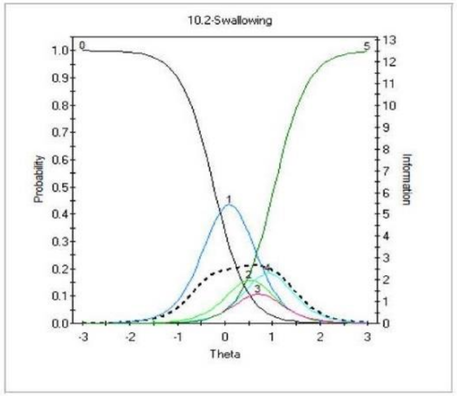
10.2-Swallowing
Probability
Information
Theta

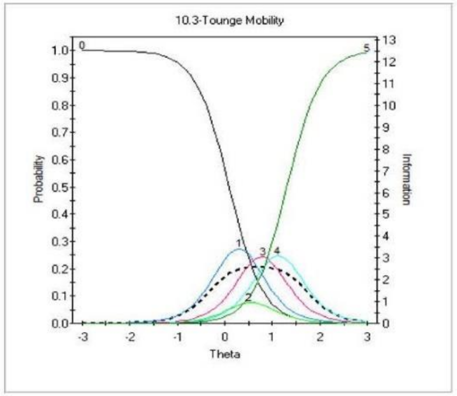
10.3-Tounge Mobility
Probability
Information
Theta

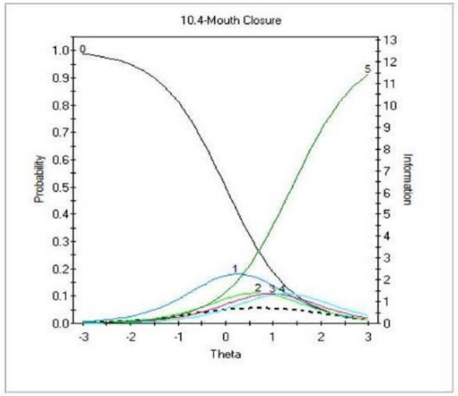
10.4-Mouth Closure
Probability
Information
Theta

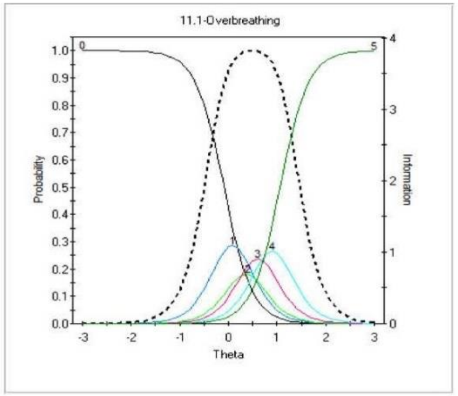
11.1-Overbreathing
Probability
Information
Theta

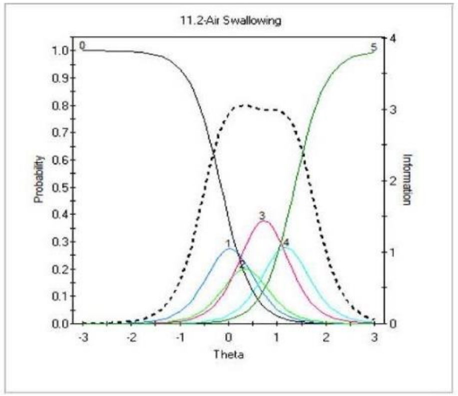
11.2-Air Swallowing
Probability
Information
Theta

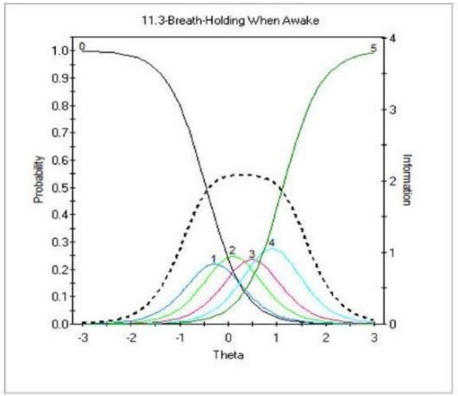
11.3-Breath-Holding When Awake
Probability
Information
Theta

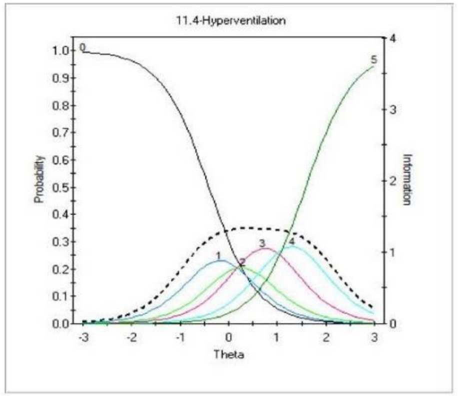
11.4-Hyperventilation
Probability
Information
Theta

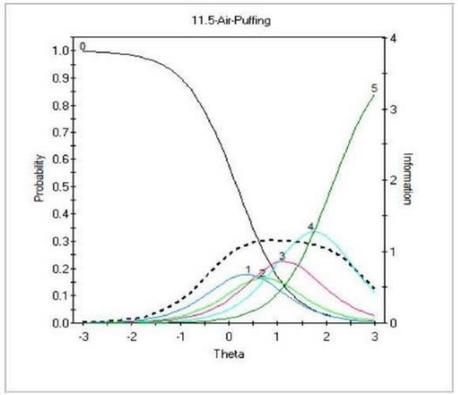
11.5-Air-Puffing
Probability
Information
Theta

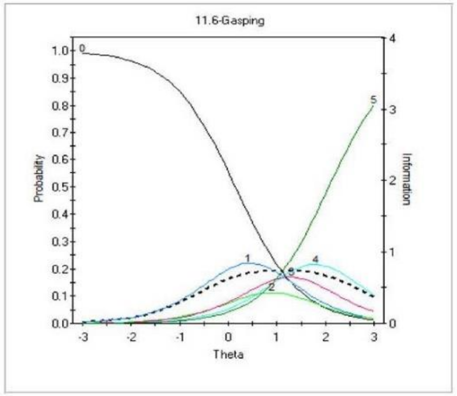
11.6-Gasping
Probability
Information
Theta

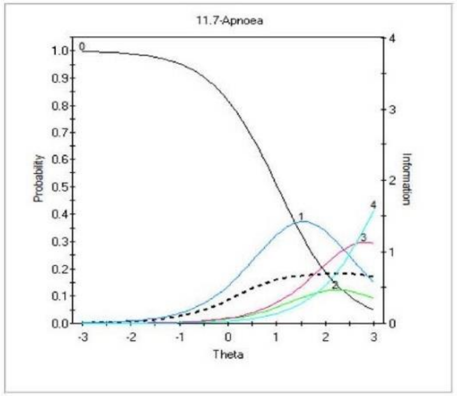
11.7-Apnoea
Probability
Information
Theta

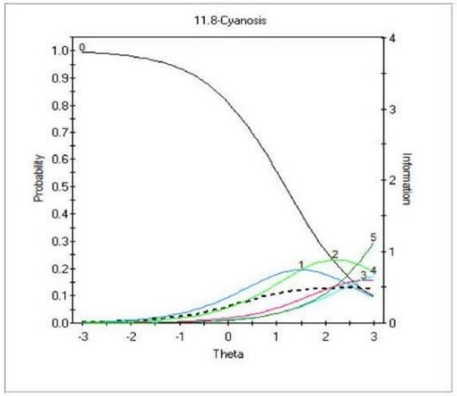
11.8-Cyanosis
Probability
Information
Theta

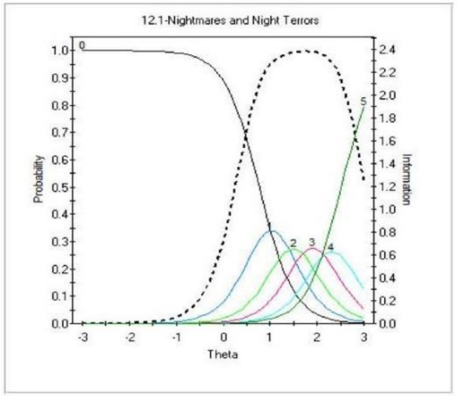
12.1-Nightmares and Night Terrors
Probability
Information
Theta

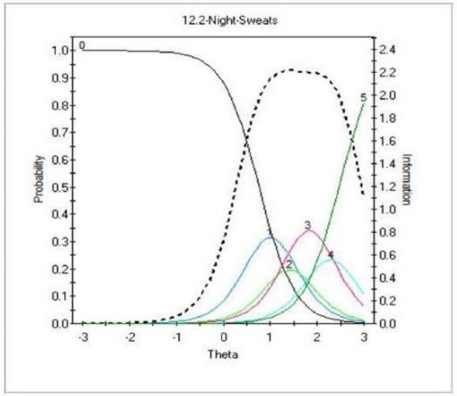
12.2-Night-Sweats
Probability
Information
Theta

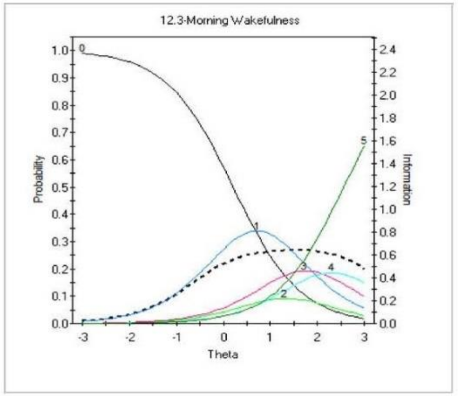
12.3-Morning Wakefulness
Probability
Information
Theta

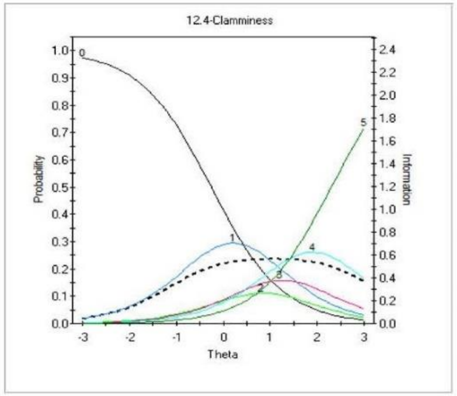
12.4-Clamminess
Probability
Information
Theta

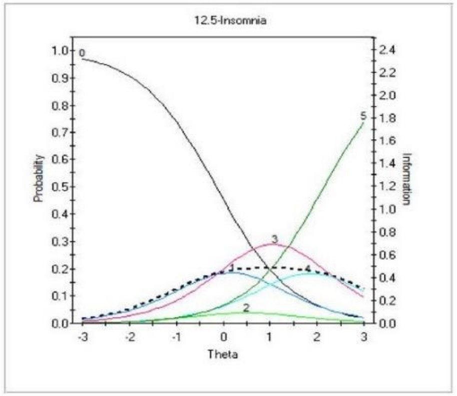
12.5-Insomnia
Probability
Information
Theta

B: Multi-System Profile of Symptoms Scale Supplement - Item Curve Characteristics

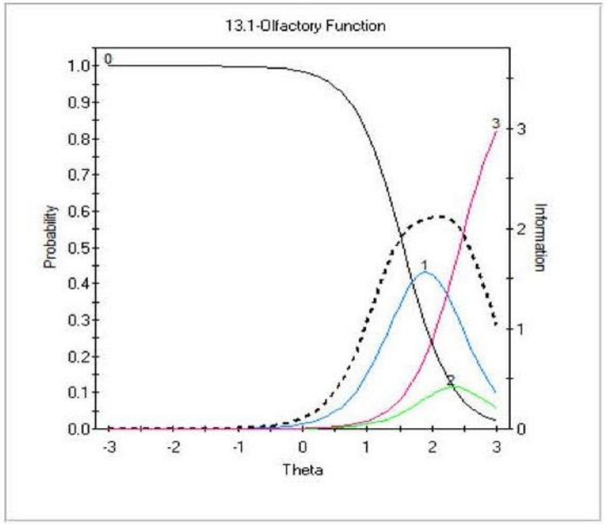

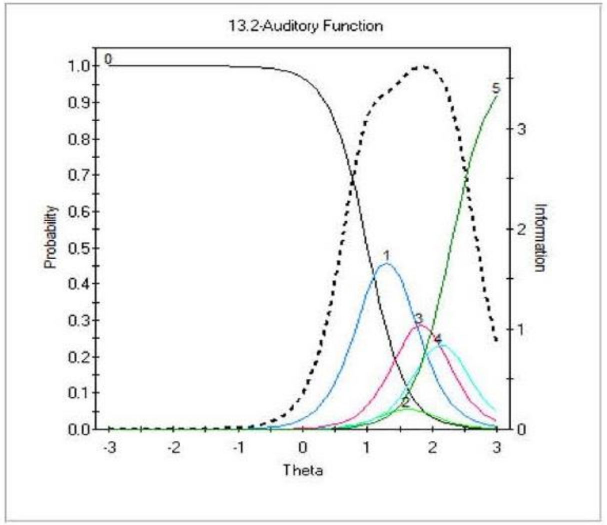

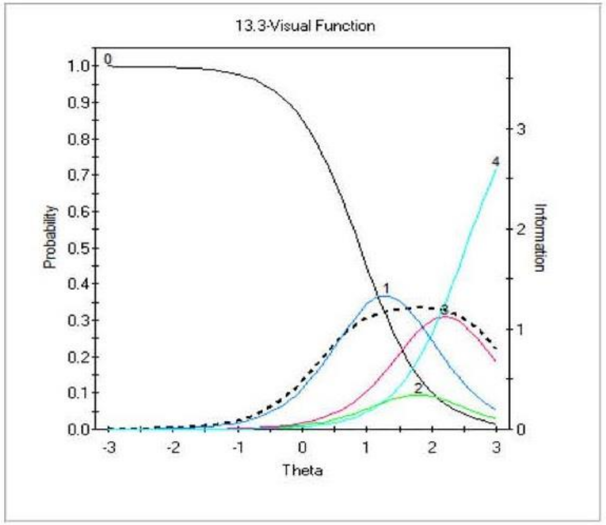

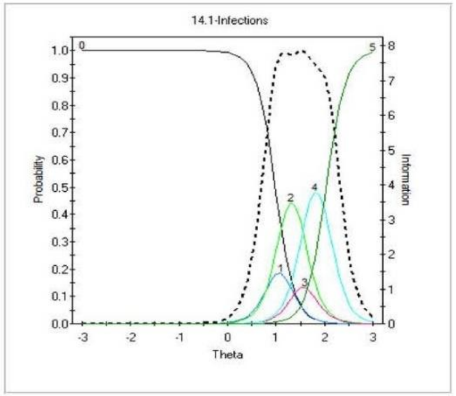
14.1-Infections
Probability
Information
Theta

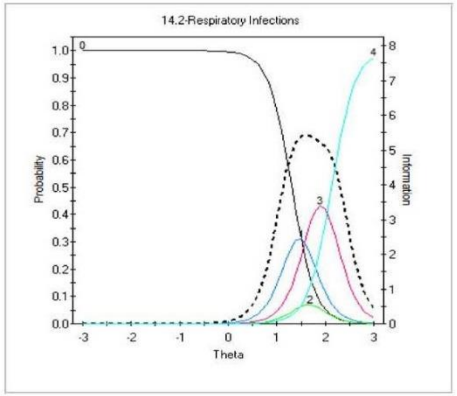
14.2-Respiratory Infections
Probability
Information
Theta

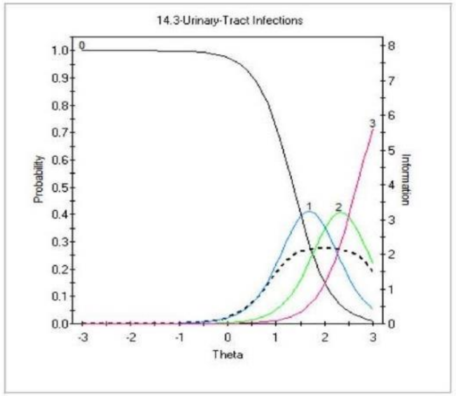
14.3-Urinary-Tract Infections
Probability
Information
Theta

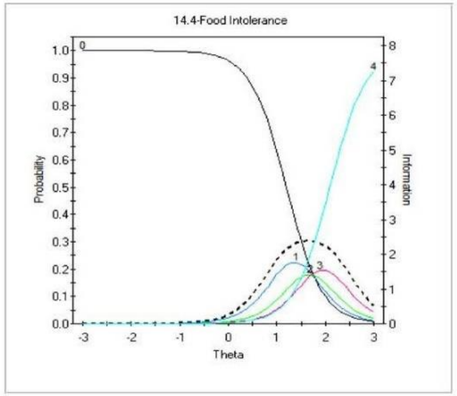
14.4-Food Intolerance
Probability
Information
Theta

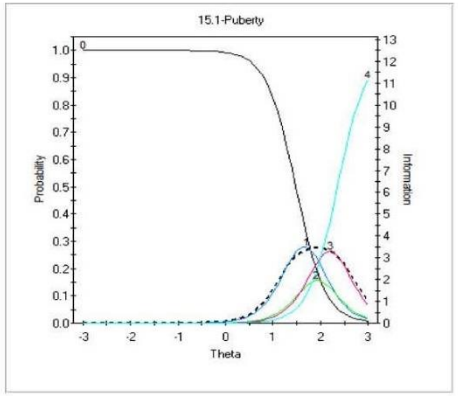
15.1-Puberty
Probability
Information
Theta

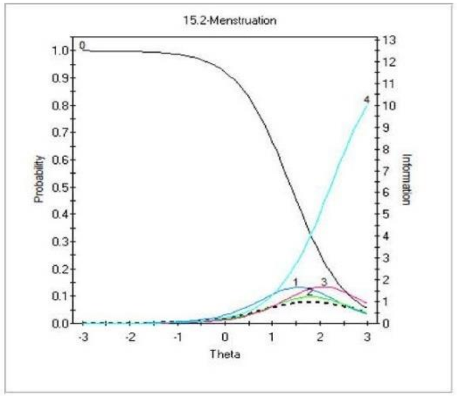
15.2-Menstruation
Probability
Information
Theta

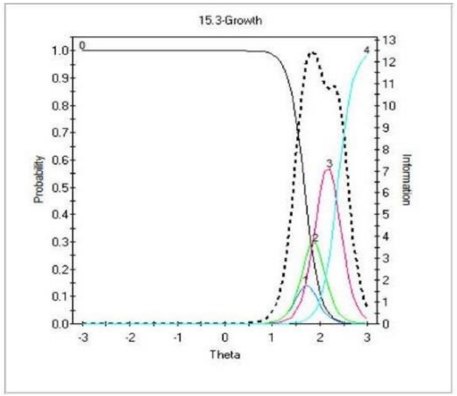
15.3-Growth
Probability
Information
Theta

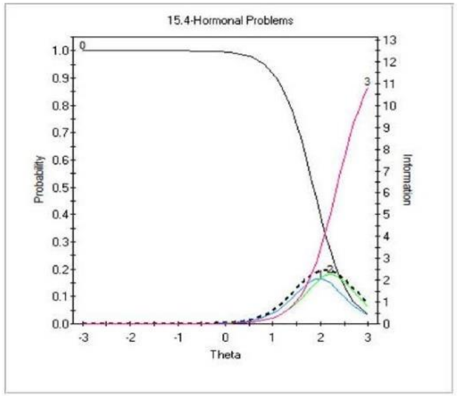
15.4-Hormonal Problems
Probability
Information
Theta

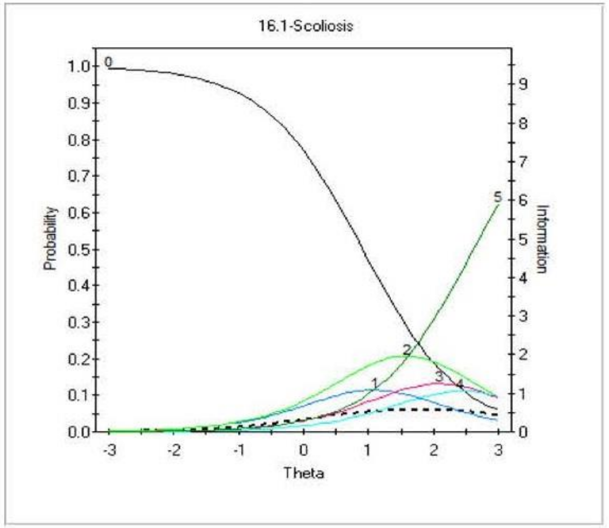
16.1-Scoliosis
Probability
Information
Theta

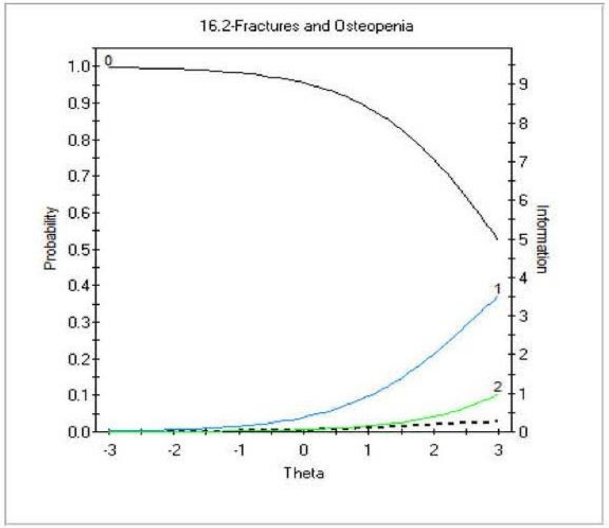
16.2-Fractures and Osteopenia
Probability
Information
Theta

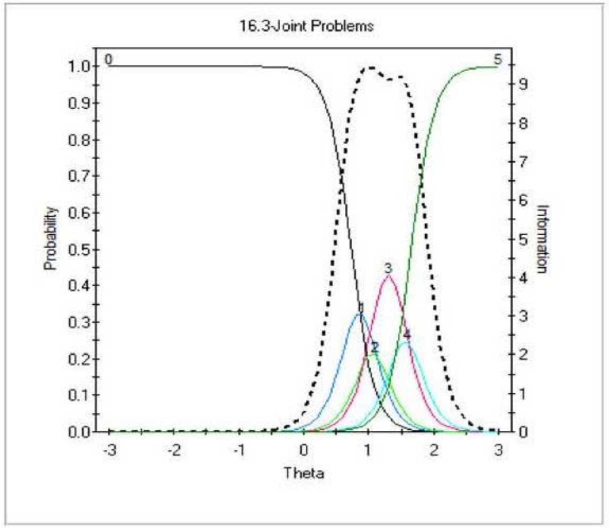
16.3-Joint Problems
Probability
Information
Theta

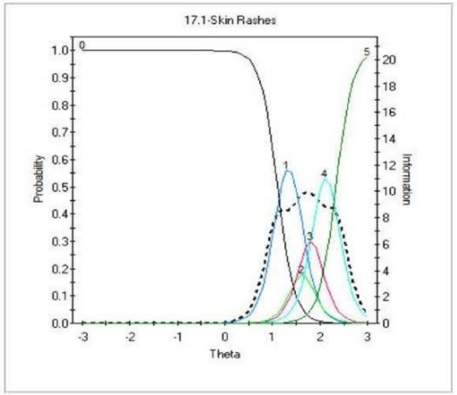
17.1-Skin Rashes
Probability
Information
Theta

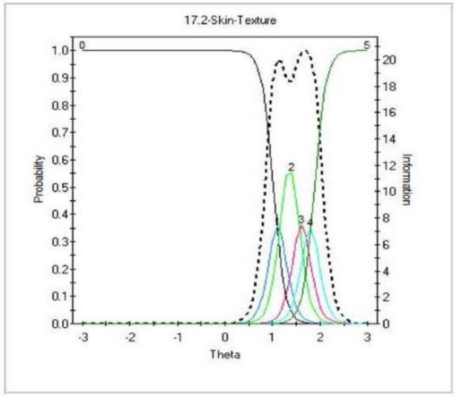
17.2-Skin-Texture
Probability
Information
Theta

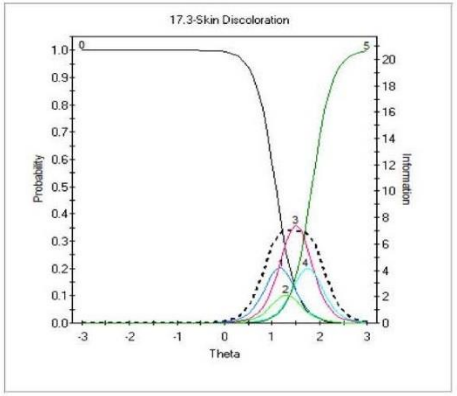
17.3-Skin Discoloration
Probability
Information
Theta

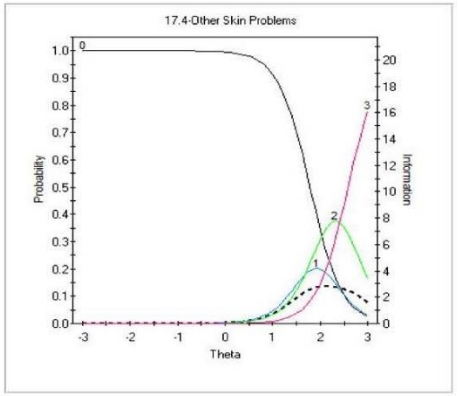
17.4-Other Skin Problems
Probability
Information
Theta
